# Understanding the gastrointestinal tract of the elderly to develop dietary solutions that prevent malnutrition

**DOI:** 10.18632/oncotarget.4030

**Published:** 2015-05-27

**Authors:** Didier Rémond, Danit R. Shahar, Doreen Gille, Paula Pinto, Josefa Kachal, Marie-Agnès Peyron, Claudia Nunes Dos Santos, Barbara Walther, Alessandra Bordoni, Didier Dupont, Lidia Tomás-Cobos, Guy Vergères

**Affiliations:** ^1^ UMR 1019, UNH, CRNH Auvergne, INRA, 63000 Clermont-Ferrand, France; ^2^ Clermont Université, Université d'Auvergne, Unité de Nutrition Humaine, BP 10448, 63000 Clermont-Ferrand, France; ^3^ Department of Public Health, The S. Daniel Abraham International Center for Health and Nutrition, Ben-Gurion University of the Negev, 84105 Beer-Sheva, Israel; ^4^ Institute for Food Sciences IFS, Agroscope, Federal Department of Economic Affairs, Education and Research EAER, 3003 Berne, Switzerland; ^5^ Escola Superior Agrária, Insituto Politécnico de Santarém, 2001-904 Santarem, Portugal; ^6^ Instituto de Tecnologia Química e Biológica, Universidade Nova de Lisboa, 2780-157 Oeiras, Portugal; ^7^ Israeli Ministry of Health, 93591 Jerusalem, Israel; ^8^ Instituto de Biologia Experimental e Tecnológica, 2780-157 Oeiras, Portugal; ^9^ Department of Agri-Food Sciences and Technologies, University of Bologna, 47521 Cesena, Italy; ^10^ UMR 1253, Science et Technologie du Lait & de l'Œuf, INRA, 35000 Rennes, France; ^11^ ainia Centro Tecnológico, E46980 Paterna (Valencia), Spain

**Keywords:** malnutrition, gastrointestinal tract, aging, dietary solutions, gerotarget

## Abstract

Although the prevalence of malnutrition in the old age is increasing worldwide a synthetic understanding of the impact of aging on the intake, digestion, and absorption of nutrients is still lacking. This review article aims at filling the gap in knowledge between the functional decline of the aging gastrointestinal tract (GIT) and the consequences of malnutrition on the health status of elderly. Changes in the aging GIT include the mechanical disintegration of food, gastrointestinal motor function, food transit, chemical food digestion, and functionality of the intestinal wall. These alterations progressively decrease the ability of the GIT to provide the aging organism with adequate levels of nutrients, what contributes to the development of malnutrition. Malnutrition, in turn, increases the risks for the development of a range of pathologies associated with most organ systems, in particular the nervous-, muscoskeletal-, cardiovascular-, immune-, and skin systems. In addition to psychological, economics, and societal factors, dietary solutions preventing malnutrition should thus propose dietary guidelines and food products that integrate knowledge on the functionality of the aging GIT and the nutritional status of the elderly. Achieving this goal will request the identification, validation, and correlative analysis of biomarkers of food intake, nutrient bioavailability, and malnutrition.

## EFFECT OF THE AGING PROCESS ON THE NUTRITIONAL STATUS

### The aging human organism

#### What does “old“ mean?

A first attempt to internationally define age was made by the World Health Organization (WHO) and United Nations declaring that “old age” is denoted by the age of 60–65 y in the developed world [1]. In particular, different gerontology experts defined further sub-groups of this population segment such as Forman et al. [2] who categorized generation 60+ in the “young old” (60–69 y), the “middle old” (70–79 y), and the “very old” (80 + y) persons or Zizza et al. [3] who divided the elderly in the three categories of “young olds” (65–74 y), “middle olds” (75–84 y), and “oldest olds” (85+ y). However, the population group of old humans is very heterogeneous and chronological age alone does not necessarily determine the physiological condition the aging organism consists of. It is, indeed, the biological age that pictures the face of aging. Every organism is growing old differently and the individual perception of this process differs depending on the attitude, living conditions, diseases and environmental influences [4]. Contingent on the state of physiological and psychological condition of the aging organism, elderly may live in different settings including acute care (hospitals), sub-acute/rehabilitation care, institutions such as nursing homes, long-term care or sheltered housing, as well as home care and free/independent living in the community [5].

#### Demographic development in Europe

The latest Demographic Report launched by the European Commission and Eurostat in 2010 starts with the words: “Older, more numerous and diverse Europeans” and highlights three main trends characterizing the current European demographic development: i) insufficient fertility, despite a slight increase, ii) longer life expectancy, and iii) important migration.

**Figure 1:**
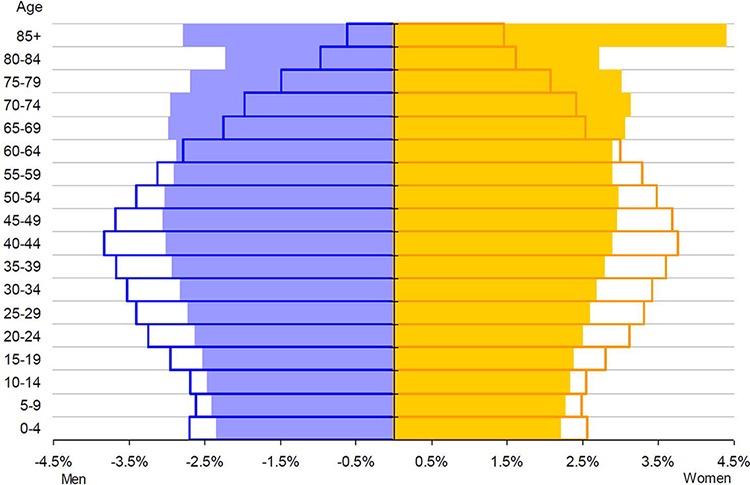
Age structure of the European population by gender and by five-year age groups [6] Each bar corresponds to the proportion of the given sex and age group to the total population. Empty bars: populations observed in 2010; filled bars: convergence scenario for 2060; blue bars (left): men; orange bars (right): women.

The age structure of populations in Europe is becoming older and this process will continue in future decades. In January 2010, the European population aged 65 y or over accounted for 17.4%. Germany had the largest proportion of this age group (20.7%), closely followed by Italy (20.2%), whereas the lowest proportion was found in Ireland (11.3%), Slovakia (12.3%), and Cyprus (13.1%). Figure 1 illustrates the structure of the European population by sex and by five-year age groups. The population pyramid of 2010 is narrow at the base and becomes more rhomboid in direction to the top. This structure is due to very high fertility rates in the mid-60ies, a time in which the baby boomer cohorts were born. The first of these large cohorts will soon reach retirement age. Furthermore, also the top of the pyramid is getting wider since longevity is increasing due to many factors such as medical progress or better supply of nutrients.

The estimated change in age structure is of higher concern than the change in population size. The proportion of the population group 65 y and older is projected to increase from 17.4% in 2010 to 30.0% in 2060 whereas most of the increase is expected to occur between 2020 and 2040. Moreover, the segment of people aged 80 y or over is growing faster than any other age group and is projected to triple by 2060. These numbers are causative of the many challenges facing the social system, health care, and politics. Time of action must be now in order to prevent these systems from collapsing [6].

#### The many faces of aging

All organs and physiological processes of the human organism are affected by aging including, *inter alia*, 1) body composition with a loss of fat-free mass (in particular skeletal muscle tissue, which is known as sarcopenia) and an increase in fat mass and its distribution [7], 2) brain function [8] with its worst outcomes dementia and Alzheimer's disease, 3) GIT function [4] with a reduction in sensory perceptions, salivation, oral health, the absorption of nutrients, and lactose tolerance, 4) fluid balance characterized by an exceedance of fluid output [9], 5) bones and joints [10] with osteoporosis and arthritis entailing falls and fractures, 6) metabolism including *e.g*. diabetes mellitus type 2 [11] and dyslipidemia [12], 7) cell growth with cancer [13], and 8) the cardiovascular system [14]. However, aging faces many changes and is not only limited to physiological restrictions as social, psychological and economic factors also strongly influence the aging process. All of these factors are currently the focus of intense research on their own. An understanding of their interactions, and a deeper knowledge of the aging consumer are clearly of upmost importance for the early identification and treatment of nutrition problems, that in turn can lead to improved outcomes and better quality of life in elderly people [15].

### The aging consumer

A large number of studies has been conducted that investigated the food consumption behavior of elderly. In particular, these studies showed that elderly feel less hungry [16], snack less often between the main meals [17], and have less cravings for food in comparison to their younger counterparts [18]. Many of these changes are related to modifications of peripheral hormones including cholecystokinin (CCK), leptin, ghrelin, insulin, and peptide YY (PYY). These hormones are released during food ingestion and play a crucial role by affecting, on the one hand, the activity in the key brain areas, which in turn control food intake [19] and, on the other hand, parts of the GIT [20]. The mechanisms by which these hormones influence food intake, satiety, and hunger are reviewed elsewhere [20]. However, other physiological factors influence food intake in elderly, in particular 1) edentulism and dental problems that impact on both oral function and social interactions [21], 2) xerostomia (“dry mouth syndrome”), which changes the perception of taste and smell and promotes caries and infections in the mouth [22, 23], 3) a reduction of olfactory, gustatory, and visual food perception leading to a decrease in appetite, a lower diversity in meal composition and food choices [20], 4) a reduction of fluid intake, which is often due to a diminished thirst sensation but also to anxiety about incontinence and toileting assistance and which may lead to cognitive and physical impairments or, in the worst case, to an increased mortality risk [9], and 5) changes in central brain control, *i.e*. in the hypothalamus, which controls hunger and satiety as well as the activity of important neurotransmitters and neuropeptides including serotonin, dopamine and opioids [24]. In addition to these physiological changes, psychological and social factors also contribute to an alteration of food intake during aging. The psychological factors include 1) depression, which is a common disorder in elderly frequently accompanied by a loss of appetite and a decrease in food consumption [25], 2) apathy, characterized by a lack of interest and emotions, which can occur as an independent disorder or as a symptom of depression or cognitive decline, and which reduces the motivation to eat and drink [20], and 3) mood, a positive or negative emotional status, which strongly influences food intake [26]. Finally, a strong contribution to changes in eating behavior is also attributed to social factors including loneliness, social isolation, widowhood, poverty, and a change of environment such as housing (nursing home, hospital, free-living…). These social changes may impact negatively on the eating habits of elderly and meals are consequently prepared less frequently, with significantly less diversity in their composition, and with a lost in the pleasure of eating [25]. This list is not exhaustive and other factors, such as income, education, diet-related attitudes and beliefs, convenience, a decreased mobility and dexterity that render the shopping and cooking more difficult, likely play a role as well in the onset of malnutrition in the elderly [27].

In summary, the aging consumer, in particular if their health status is weakened, is influenced by many factors whose relationships are not yet fully understood. The occurrence of already a few of these factors may therefore impact on the others and strongly increase the risk of malnutrition.

### Malnutrition in aging

#### Definition and causes of malnutrition

Malnutrition is one of the most relevant conditions that negatively influence the health of older people and the nutritional status of elderly (65 + y) was even recently shown to predict preterm death [28]. Although aging is not inevitably accompanied by malnutrition, many changes due to the aging process can promote this serious condition [29].

Since there is no official definition of the term “malnutrition”, different organizations and groups working in this field described malnutrition as the state of being poorly nourished, due to a lack of one or more nutrients (undernutrition) or an excess of nutrients (overnutrition) [30]. WHO states that malnutrition is the cellular imbalance between supply of nutrients and energy and the body's demand for them to ensure growth, maintenance, and specific functions [31]. However, the main concern is rather undernutrition than overnutrition because its relation to morbidity and mortality is much stronger than that of obesity [32]. Therefore, literature mainly refers to undernutrition when addressing the topic of malnutrition.

The main cause for malnutrition is a reduced and/or unbalanced dietary intake. However, this reduced intake can be due to many factors that are again divided in three main categories: social, physiological, and psychological. Examples for each category are summarized in Table 1 [30, 33, 34].

**Table 1:** Causes of malnutrition

Social factors
Lack of knowledge about food, cooking, and nutrition
Isolation/loneliness
Poverty
Inability to shop and/or prepare food
Inability to prepare food
Physiological factors
Gastrointestinal dysfunction, e.g. malabsorption
Poor appetite and poor diet
Oral problems such as teeth loss and dysphagia
Loss of taste and smell
Respiratory disorders
Endocrine disorders, e.g. diabetes mellitus type 2
Neurological disorders, e.g. Parkinson disease
Infections, e.g. urinary tract infections
Physical disability to feed self
Drug interactions
Nausea and vomiting
Altered/increased metabolic demands
Other diseases, e.g. cancer
Psychological factors
Dementia
Depression
Confusion
Anxiety

Note: see references [30, 33, 34]

#### Screening tests for malnutrition

Many attempts have been made in order to develop efficient nutritional screening and assessment tools that detect malnutrition in humans living in a variety of settings. The establishment of these tools is critical in order to diagnose in an early stage and, eventually, prevent malnutrition. The parameters that are essential components of malnutrition screening include a reduced dietary intake, weight loss, a reduced nutritional status, and the existence of diseases [35]. A variety of screening tests have been developed among these the Nutritional Screening Index, SCREEN, the Appetite, Hunger and Sensory Perception Questionnaire (AHSP), the Malnutrition Universal Screening Tool (MUST), and the Saint Louis University SCALES nutritional index. The screening of malnutrition also includes a range of anthropometric parameters such as BMI, arm span, waist circumference, or mid-arm circumference measurements. Each of these tests varies with regard to the type of data collected, their specificity and their sensitivity. Importantly, no single test is available that optimally detects malnutrition in a broad range of health conditions (healthy vs non-healthy elderly) and housing settings [36].

The most frequently used tool for determination of the nutritional status in aged people is the mini nutritional assessment (MNA) [5]. MNA was first developed by Vellas and Guigoz approx. 20 years ago [37] to be then continuously improved over the years. MNA is currently the gold standard of nutritional assessment since it is adapted to the older age group, relatively easy to use, and highly sensitive [5]. MNA consists of 18 questions grouped in four categories: anthropometry, general status, dietary habits, and self-perceived health and nutrition states. The summation of the scores awarded to the different questions (maximum 30 points) allows a grading of the nutritional status (score > 24 points: good status, 17 < score < 24 points: risk of malnutrition; score < 17 points: malnutrition). A shorter form of MNA, MNA short form (MNA-SF), was developed in 2001 [37] and validated in 2009 [38]. MNA-SF also divides the nutritional status in three categories but the time for completion is significantly reduced and the scoring is independent of BMI. However, due to its low specificity, MNA has been associated with a high risk of “overdiagnosis” [5]. Consequently, experts recommend, particularly for unclear cases, to complement MNA with other screening tools. These experts also advocate the need for further research in that field [5].

#### Prevalence of malnutrition and impact on costs

Malnutrition occurs in all residential and living settings [39]. The estimates of its prevalence are highly variable due to the use of different evaluation tools and different settings. Previous publications reported prevalence ranging from “almost non-existing” in healthy, community-living “young elderly” [40] to 57% in persons living in long-term care institutions [41]. Furthermore, the prevalence differs between rural (7.4% malnourished) and urban (18.5% malnourished) living individuals [42]. In 2010, Kaiser et al. [39] published a review that more precisely determined malnutrition among older adults (65 y and older). The reviewed studies were selected from the literature if MNA was used and if the setting (community-dwelling, nursing home, hospital, geriatric rehabilitation) was clearly described. The effective sample size was 4,507 (24 studies from 12 countries, mainly of European origin) with an average age of 82.3 y. In total, 46.2% of the participants were at risk of malnutrition and 22.8% were malnourished. When only focusing on the two groups of elderly that were hospitalized or in geriatric rehabilitation, approx. 90% of them were malnourished or at risk of malnutrition. In the nursing home setting, only one third of participants were well nourished. Furthermore, 31.9% of elderly living in the community were at risk of malnutrition whereas only a small fraction was malnourished. Another review published in 2011 investigated the worldwide prevalence of malnutrition and the risk of malnutrition in the elderly population [5]. For this analysis, the data was extracted from published and unpublished studies in which the nutritional status was measured with MNA or with a validated MNA-SF and the living setting was clearly defined. Among the elderly treated in acute care in hospitals (n = 17,775), 23.4% were malnourished and 49.4% were at risk of malnutrition. In subacute rehabilitation care (n = 3,724) malnutrition occurred in 31.0% of the individuals and 54.0% were at risk of malnutrition. In institutions, such as nursing homes, long-term care and sheltered housing (n = 20,410), 27.2% were malnourished and 52.1% were at risk of malnutrition. Also, 7.7% of elderly in home care or outpatients (n = 12,386) were malnourished and 39.6% were at risk of malnutrition. Finally, only 4.2% of community-dwelling elderly (n = 50,957) were malnourished and 27.4% were at risk of malnutrition. Of note, three of the reviewed studies were conducted in rural communities of developing countries, which reported significantly higher prevalence of malnutrition and elderly at risk of malnutrition.

In a review from the British Association for Parenteral and Enteral Nutrition (BAPEN) meeting held in 2013 malnutrition was reported to cost £ 7.3 billion per year in Britain. Malnutrition affects 10% of the population over the age of 65 y and over half of the health care costs is in this age group [43]. Using a multivariate logistic regression model Isabel et al. [44] concluded that malnutrition is an independent risk factor for a range of hospital parameters including length of hospital stay, complications, mortality, and costs. This analysis showed that malnourished patients represented a mean daily expense of US$ 228 compared to US$ 138 per well-nourished patient (increase of 60% in costs due to malnutrition). When the costs of medications and tests were added the costs of the malnourished patients rose by 309% compared to the well-nourished patients. Of note, the actual costs may vary between countries due to differences in health systems. In a study performed by Ben-Gurion University, the number of hospitalization days was doubled in elderly patients at risk of malnutrition when compared to elderly patients with a normal nutritional status. Also, significantly higher rates of readmission were observed in elderly patients at risk of malnutrition following discharge from acute hospitalization [45].

#### Strategies to prevent and treat malnutrition

Strategies that aim at treating or even preventing malnutrition in elderly are subject to intense research activity. The multifactorial character of malnutrition in aged people demands the development of holistic dietary strategies and recommendations including 1) social interferences aiming at the avoiding and revoking of social isolation as well as a monitoring of the living standard of the elderly by family members and friends, 2) the administration of nutritional supplements, 3) an improvement of the quality of care settings, and 4) in worst cases, clinical interventions. However, the core of the multi-disciplinary strategy to prevent and treat malnutrition in the elderly is undoubtedly the dietary intervention as advocated by the “first food policy” whose major objective is to ensure adequate supply of food to all [46, 47]. To be successful, the intervention requires more detailed evidence-based dietary recommendations for elderly and novel food products, which suit the needs and requirements of elderly. The fourth part of this review will present this topic in detail.

Malnutrition affects the function and recovery of every organ system in humans: it impairs liver, gut and renal function, wound healing, decreases immunity and muscle strength as well as cardiac output, and moreover may cause depression and apathy [33]. A poor nutritional status is a major negative prognostic indicator in the elderly population [48, 49]. Regardless of BMI or weight loss caused by undernutrition in persons aged 60 + y malnutrition is also associated with increased mortality [36]. Furthermore, malnutrition is associated with longer hospital stays, re-admission, immune dysfunction, high demands on medical services, and early institutionalization. Moreover, a higher risk of chronic disability such as frailty and poor quality of life are further serious consequences of malnutrition, more precisely undernutrition [34]. Finally, malnutrition has emerged as an important factor in the development of sarcopenia and dementia [50]. The third part of this review will present this topic in detail.

Malnutrition increases the risk for frailty in elderly, and in turn the aging process increases the risk of malnutrition. To break down this negative loop, with the final aim of maintaining good health and a high quality of life in the ageing population by providing them a balanced diet taking into account their nutritional needs, an in depth analysis of the impact of age-related changes on the nutritional status is needed. In the past, research focused on the cognitive decline as well as on the changes taking place in body composition and organ function as age progresses. Although the GIT is crucial for the release and delivering of nutrients from foods to the human body an exhaustive analysis of phenomena occurring in the GIT during aging and on their impact on malnutrition has never been conducted. The second part of this review will address this particular point.

## THE AGING GASTROINTESTINAL TRACT

### Effect of aging on GIT functions

#### Chewing activity

The mechanical and chemical processes of digestion start in the mouth, with mastication. This first step is needed for the mechanical breakdown of food into smaller particles and is assisted by saliva secretion for fragments lubrication, moistening, and initiation of oral digestion. A large contact area between food and saliva is important in order to form a cohesive food bolus ready to be swallowed. Saliva also initiates digestion through the action of salivary enzymes, such as alpha-amylases or lipases, which help break down the chemical bonds in food constituents [51]. The two actions of mastication and salivation, under a permanent adjustment of the masticatory forces to the food properties [52], coordinate to form a bolus whose consistency progressively reaches the structural properties needed to ensure a safe-swallowing. Apart from food structural disruption, chewing also participates in the release of sensory signals involved in taste perception.

The main age-related changes in the oral sphere are a decrease in bite force and mandibular reflex occurrences, a decrease in the number of oro-sensory receptors (mechano- and gustative receptors) leading to an increase in sensory thresholds, and a decline in saliva secretions [53, 54]. Motor activity of tongue and masticatory muscles also declines.

Masticatory function in elderly depends on two major factors, which are the number of natural antagonist teeth and the quantity and quality of saliva. Subsequent potential nutritional consequences are generally considered according to two different viewpoints [55, 56].

The first viewpoint considers a healthy oral aging taking place without any important oral disorders such as tooth loss or critical saliva deficiency. For this segment of population, aging is associated with a decrease in maximal bite force [57] and changes in masticatory muscle tissue [58, 59]. However, these alterations have little impact on masticatory performance as these persons still produce a food bolus suitable for swallowing and only minor adaptations are needed to compensate the physiological changes [60, 61]. In particular, the number of masticatory cycles needed to form the food bolus increases with age (three additional masticatory cycles every ten years) [62]. Consequently, the masticatory process before swallowing is lengthened and the total masticatory muscle contraction is increased without altering the resulting food breakdown [55, 62–64]. Concerning swallowing, which ends the masticatory process, the major change occurring with age under healthy conditions appears to involve modifications in the temporal cascade of events to adapt to a slight decrease in oral perception of viscosity, rather than to adapt to a decrease in swallowing reflex [65, 66].

The second viewpoint considers the impact of oral functioning on digestion or nutritional status in the elderly [67]. Elderly with a good oral health maintain their potential of adaptation of masticatory parameters with little or no consequences on subsequent digestion. Another segment of the elderly population suffers, however, from poor oral health, which is characterized by a high prevalence of tooth loss and oral disorders related or not to other systemic diseases. In this population group, adaptation of the oral process becomes less efficient, even fails, and finally leads to an impaired function. Elderly tend to have fewer natural teeth and higher rates of tooth loss until edentulism [68]. Tooth loss causes impaired mastication and tissue alterations in the mandibular bone, and the level of resulting impairment is linked to the number of remaining teeth [69]. The degree of food size reduction, reflecting chewing efficiency or performance, is greatly reduced in denture wearers in a gradual manner depending on the number of teeth lost [55, 70]. Numerous studies showed that elderly with a compromised dentition fail to prepare a food bolus that is ready for swallowing because an insufficient disruption of the bolus is associated with a greater proportion of large particles [55, 63, 71, 72]. A median value of 4 mm particle size of a bolus of raw carrot defines the swallowing threshold, which is used to conclude if mastication is correct or not [73]. The consequences of an insufficient disruption of the food bolus are worsened if this phenomenon is accompanied by a lack in saliva, as frequently observed in the elderly, especially those under medication, which is known to affect salivation [74]. Apart from an insufficient breakdown of food, scarcity of saliva during bolus formation also increases the risk of dysphagia and aspiration of food fragments, impairs early digestion and the dissolution of nutrients [75]. In addition, denture wearers fail to adapt to changes in food texture such as hardness [55]. Importantly, an impaired oral health often leads elderly to modify their diet to adjust it to their limited oral functional capacities [76]. These changes are mostly not adequate to maintain good overall health since soft foods are often foods rich in fat and contain additives [77].

Masticatory deficiency seems to be a risk factor for cognitive dysfunction [78]. Although a clear causal evidence between malnutrition and poor oral health in the elderly population is still missing, it appears evident that elderly suffering from oral problems are exposed to an increased risk of malnutrition, either by avoiding nutritious food being difficult to chew or by swallowing food boli that are insufficiently disrupted for a thorough assimilation of the nutrients [75, 79, 80]. Based on a systematic review of several published works, van Lancker et al. [81] found an association between malnutrition and the oral health status, although the main cause of malnutrition was a reduced intake of foods of good quality. Future research is needed to investigate if causal relationship exists.

Meanwhile, some oral health indicators must be included into nutritional studies in the elderly population. As already suggested by Hatch et al. [82], the number of functional units is a key predictor of masticatory performance. In the same line, El Osta et al. [83] proposed to consider the perception of xerostomia, the number of functional units present in the mouth, and the score obtained with the Geriatric Oral Health Assessment Index (GOHAI) [84] as the most appropriate oral health indicator. Such an evaluation of oral health could be combined with the MNA to reliably identify elderly at high risk of malnutrition [85].

#### Food transit in the different GIT segments, and motor activity

##### Food transit

###### Stomach

Gastric emptying plays a key role in the kinetics of nutrient absorption, which in turn regulates nutrient utilization in body functions. This is clearly illustrated by the concept of slow/fast carbohydrates and proteins and their respective effects on glucose and protein homeostasis. Mechanisms involved in food disintegration and gastric emptying have been well described in the review of Kong and Singh [86]. Liquid and solid meals display different gastric emptying rates after ingestion. The halftime, t_1/2_, indicating when 50% of ingested meal is emptied, ranges from 10 to 60 min for liquid meals [86], whereas t_1/2_ reported for solid foods ranges from 50 min (bread and noodles) to 115 min (beef liver) [87–90]. Gastric emptying rate is influenced by other meal components [87], meal volume [91], caloric content [92], the ratio between liquid and solid in the meal [93], and the type of dietary fibers [94]. Furthermore, for solid food, chewing efficiency and the degree of disintegration of the swallowed bolus affect gastric emptying rate [90] and, consequently, the kinetics of nutrient absorption [95].

Conflicting data are reported in the literature regarding the effect of aging on gastric emptying rate. Some studies reported no significant effect [96, 97] whereas others reported only a trend toward prolonged gastric emptying of solids and slightly faster emptying of liquids [98, 99], the increase in liquid outflow from the stomach being explained by a reduced compliance of the antrum [99]. Finally, some studies observed a significant increase of 30–40% in solid and liquid gastric emptying time in the elderly [100–102]. Consequently, no clear conclusions on this research area can be made, mainly because of the variability of measurements and strongly differing health states in the elderly population. In this context, a study divided this population in frail and non-frail elderly and showed that, compared to young adults, gastric emptying time significantly increased in frail elderly, whereas it was unchanged in non-frail elderly [99].

###### Small bowel

Small bowel transit time in young adults ranges from 2 to 6 h [97, 103]. The effect of aging on small intestinal motility is not very well documented in the literature. Although the propagation velocity of phase 3 of the migrating motor complex is slower in the elderly, the patterns of motility and the transit rate appear to be maintained in the small intestine during aging [97, 104–106].

###### Colon

Few studies specifically investigated the effect of aging on colonic transit time. Metcalf et al. [107] reported no significant effect of aging on the transit time in the different segments of the colon whereas Madsen and Graff [97] evidenced a significant increase of colonic transit time in older subjects (+70%). Of note, environmental factors, such as physical inactivity [108], can largely increase colonic transit time. These confounding factors make it difficult to conclude on the specific role of aging. Of note, the prevalence of constipation increases with age. 30–40% of community-dwelling older adults and over 50% of nursing home residents experience chronic constipation. However, constipation does not seem a physiological consequence of normal ageing and the underlying reasons for constipation in advanced age include insufficient fluid and dietary fiber intake, reduced physical activity, age associated diseases, and chronic medications [109].

In conclusion, the effect of aging segmental transit time in the gut has not been sufficiently investigated and conflicting results do not allow to make clear conclusions. Transit time could be prolonged in the stomach and the colon. This effect seems weak for the general population, but could be significantly more pronounced in elderly with masticatory deficiency, reduced physical activity, and frailty syndrome. The diversity of experimental approaches measuring regional transit time also explains result inconsistency. These methods include mainly scintigraphy, radio-opaque markers, ultrasonography, breath tests, and paracetamol test, the last three methods being specific for the stomach. In addition, the use of wireless motility capsule is developing. This technology can provide information on intraluminal pH and pressure, is cheap and ambulatory, and the data correlates well with scintigraphy [110]. Wireless motility capsule is thus a very interesting tool to increase knowledge on the evolution of the regional gut transit during aging.

##### Motility and its regulation

Although gut motility seems impaired in older subjects, it is not clear whether this impairment is directly linked to a decreased ability of smooth muscles to contract and relax or to alterations in the regulation of these movements by the enteric nerves or specialized cells such as the interstitial cells of Cajal. At the level of the smooth muscle itself, impairment in signal transduction of the phosphorylation of the light chain of myosin [111] as well as perturbation of calcium signaling [112] have been evidenced in the colon. Few studies have specifically addressed the effect of advanced age on enteric nervous system. Hanani et al. [113] observed a significant change in the morphology of the myenteric plexus of the human colon (increase in cavities) as a consequence of aging, but this observation was mainly driven by the increase in the cavities observed between 0–25 y, variations between 50–90 y being much less significant. However, supporting the hypothesis of an increase in abnormal myenteric ganglia in elderly, Bernard et al. [114] evidenced a neuronal loss in the myenteric plexus, this loss being specific to the cholinergic subpopulation, whereas nitrinergic neurons were spared. In animal models, the decrease in the number of neurons in advanced age is controversial [115]. However, signs of neurodegeneration have been clearly observed, as indicated by swollen and dystrophic nerve fibers, lipofuscin accumulation, and protein alpha-synuclein aggregates accumulation [115, 116]. The accumulation of aggregates could be linked to deficiency in macrophage and proteolytic activity [116, 117]. Studies in human elderly failed to show significant changes in neuron number in the submucosal plexus [114, 118]. However, a study in rodents showed that a decline in the number of neurons could be very progressive and specific to the distal colon plexus [119]. For networks of interstitial cells of Cajal, which contribute to segmenting and peristaltic contractile activity, a decline in density and volume has been observed in the colon of elderly subjects [120, 121].

In conclusion, more than a reduction in number, the degeneration of neurons and glia may partly explain modifications in GIT motility during aging.

#### Food digestion

Food digestion is ensured by different enzymes secreted in the first part of the digestive tract (from mouth to duodenum) and by microbial digestion in the hindgut.

##### Digestion by endogenous enzymes

###### Saliva

Saliva contains alpha-amylase involved in polysaccharide digestion. Conflicting data has been reported regarding the effect of advanced age on salivary flow. The latest study in this area showed a significant decline (−50%) of saliva in elderly [122] independently of medication. In agreement with this finding, various morphometric and histopathological changes have been described in old mice [123]. However, despite the decrease in secreted saliva volume, a greater daily alpha-amylase output has been observed in advanced age [124]. Some lipolytic activity has been detected in oral cavity; however it is weak and only involved in oro-sensory fat detection [125, 126].

###### Stomach

In healthy elderly, gastric acid secretion was reported to be either unaffected [127–130] or increased [131]. However, *Helicobacter pylori* infection and atrophic gastritis are both associated with a decline in gastric acid secretion and their prevalence increases with age. Regarding pepsin, both basal and stimulated secretions decline after 70 y (divided by four between 70 y and 90 y) and this decline is independent of atrophic gastritis and *H. pylori* infection [128].

###### Pancreas

Studies in animal models showed a decrease in pancreatic secretions in advanced age [132–134]. In particular old animals are unable to adapt their pancreatic exocrine secretion to changes in dietary intake. Three studies conducted in humans, in which pancreatic exocrine secretions were recorded by duodenal collection, showed that, compared to young controls, subjects above 65–70 y had significantly reduced bicarbonate and enzyme (lipase, chymotrypsin, amylase) secretions, due to both a decrease in secreted volume and enzyme concentrations [135–137]. In agreement with these findings, magnetic resonance imaging evidenced an increase in pancreatic atrophy, lobulation, and fatty degeneration during aging [138]. Using fecal elastase-1 as a marker of pancreatic exocrine dysfunction, a large population-based study (50–75 y) reported a clear increase in exocrine pancreatic insufficiency with aging [139]. The same observation was made in a population of persons older than 80 y having no factors known that increase the prevalence of pancreatic deficiency, in particular gastrointestinal disorder, surgery, or diabetes mellitus [140]. Using strict selection criteria that ensured a very good health status of the included subjects, Gullo et al. [141] observed no significant impairment of the pancreatic function in very old subjects (> 91 y). In conclusion, the pancreatic exocrine secretion does decline in advanced age. This decline may, however, not be sufficient to cause maldigestion. Despite advanced age, the number and mass of β-cells are relatively well preserved in the endocrine pancreas of nondiabetic individuals, compared with the exocrine pancreas [142]. Age-related impairment of pancreatic beta-cell function has been reviewed by De Tata [143] and will not be described here. Of note, however, ageing is clearly associated with a decline in insulin action (insulin resistance), resulting in higher fasting and postprandial glucose concentration [144].

###### Bile

Biliary secretion does not contain enzymes and, thus, is not directly involved in digestion. Nevertheless, bile contains bicarbonate, which helps in the neutralization of chyme pH, and biliary salts, which are involved in fat absorption. Both fasting and maximally contracted gallbladder volumes are not affected in old age [145, 146]. Similarly, the secretion of total bile acid seems not to be affected by aging [145, 147]. However, bile acid reabsorption could be impaired in elderly [145].

#### Microbial digestion

A reduced biodiversity and compromised stability of the intestinal microbiota is often observed in elderly when compared to younger subjects [148]. The effect of aging on Firmicutes and Bacteroidetes, the two dominant divisions of the gut microbiota, and their ratio is controversial [149]. At a lower phylogenetic level of the microbiota, facultative anaerobes, including opportunistic proinflammatory bacteria, increase in advanced age, whereas health-promoting bacteria, such as Bifidobacteria, seem unaffected [149]. The composition of the microbiota of elderly significantly correlates with measures of frailty, co-morbidity, nutritional status, and markers of inflammation [150]. The effect of age-related microbiota changes on the digestive function of the colon is, however, less documented. Functional metagenomics showed that the age-related trajectory of the gut-microbiome is characterized by loss of genes for short-chain fatty acid production and an overall decrease in the saccharolytic potential, while the proteolytic potential seems to increase [151]. In line with these observations, a lower colonic fermentation has been observed in elderly women, compared to young women, after ingestion of a test meal [106].

#### The gut wall

##### Mucosal turnover

The epithelium of the GIT undergoes constant and rapid renewal (every 2 to 6 days according to the gut segment and diet). After removal of the confounding effect of diet, animal models showed that the architecture of the epithelium (such as villus height and crypt depth) is globally unaffected in advanced age [152, 153]. Similarly, no morphological changes of the duodenum were observed in elderly subjects [154]. A hyperproliferative state balancing an increased rate in enterocyte apoptosis was proposed to account for this lack of morphological change [155]. The total surface area available for absorption in the small intestine is therefore not deeply affected by aging. The increase in cell proliferation is accompanied by a rise in expression and activation of several tyrosine kinases, including the epithelial growth factor receptor (EGFR). The increase in EGFR activation with age could be linked to a decrease in the EGFR-related peptide, a negative regulator of EGFR [156]. In contrast to the data reported for the human duodenum, studies in the colon of rodents showed an increased mucosal cell proliferation accompanied by decreased apoptosis. This decrease could be explained by decreases in ‘cell cycle and apoptosis regulatory protein-1′ (CARP-1) that participates in EGFR-dependent signaling [157].

##### Permeability

On the basis of the lactulose/mannitol test, which delivers a permeability index, the tightness of the small intestine barrier appears to remain intact with advancing age [158, 159]. The situation could be different at the level of the colon as a study in non-human primates found a higher intestinal permeability in aged animals, in connection with a remodeling of tight junction proteins [160].

##### Carrier function

Using an *ex-vivo* approach in an aging rodent model, Woudstra et al. [161] did not observe age-related quantitative changes in lipid uptake (per unit of mucosal surface area), ileal lipid-binding protein (ILBP), and the cytosolic fatty acid binding protein (FABP). However, in a mice model, a significant increase in cholesterol absorption was observed, in line with an increased biliary cholesterol output, and an up-regulation of the expression of the influx transporter NPC1L1 in the different segments of the small intestine [162]. One study reported a complete profiling of the transporters involved in fatty acid (FATP4, FABPpm, FAT/CD36) and cholesterol absorption (NPC1L1, ABCG5/ABCG8, ABCA1) in humans aged 37 y to 83 y [163]. However, the sample size of the study was limited (n = 11) and the authors did not find any decrease in lipid transport proteins with aging.

In contrast to proteins, the uptake of sugars, expressed on the basis of mucosal surface area, appears to be influenced by age as fructose uptake was reported to increase in advanced age whereas glucose uptake declined [164, 165]. These modifications were, however, not explained by variations in the transporters GLUT2, GLUT5, or SGLT1. The effect of aging on intestinal amino acid and peptide transporters has not been investigated in humans. However, a study in mice suggested that amino acid uptake by intestinal wall is not affected by aging [166].

Calcium absorption relies on both passive and active uptake processes through the enterocytes. The main proteins involved in active calcium absorption are the calcium-binding protein calbindin-D_9k_, the luminal channel transient receptor potential vanilloid 6 (TRPV6), and the plasma membrane Ca^2+^-ATPase (PMCA1b). The expression of these proteins declines in advanced age [167, 168]. Furthermore, tight junction proteins (claudin-2 and claudin-12) could also facilitate paracellular calcium transport [169] but the effect of aging on this pathway is not known. Many proteins involved in active- or facilitated calcium absorption are upregulated by 1α,25-dihydroxyvitamin D_3_ (1,25(OH)_2_D_3_), whose ability to stimulate calcium influx markedly decreases with age. Possible causes involved in the modification of the 1,25(OH)_2_D_3_ response with age have been reviewed by Gonzales-Pardo and Russo de Boland [170].

Heme and non-heme iron are absorbed in the small intestine by separate mechanisms. The uptake of inorganic iron in the brush-border of duodenal enterocytes relies on the divalent metal transporter DMT1, whereas heme-iron uptake is mediated by a heme transporter. Iron is then stored in the cytoplasm as ferritin and the basolateral export of iron (Fe^2+^) is mediated by ferroportin [171]. The effect of aging on the expression of the proteins involved in iron absorption is not documented.

##### Intestinal barrier and immune system

The impact of aging on the intestinal barrier and immune system has been recently reviewed by Man et al. [172]. The first actors in the barrier function of the intestine are the anti-microbial peptides (AMPs) that are secreted by epithelial cells and the mucus layer, which covers the epithelium. Whether aging impacts on AMPs is not known. Apart from *H. pylori* positive subjects, the thickness of the mucus layer is not altered in the elderly [173]. The impact of aging on the chemical composition and structure of the mucus layer is not documented. However, modifications of the mucus could explain the reduced ability of bifidobacteria to bind to the mucosa of elderly [174].

Aging is associated with a progressive decline of the mucosal immune response in the intestine, a process coined with the term “immunesenessence”. The production of antigen-specific immunoglobulin A, which is a key function of the mucosal immune response, decreases in elderly persons [175]. The ability of the aging immune system to generate tolerance to harmless antigens is also reduced [176]. Whereas the age-related changes in the systemic immune response are well documented, much less is known about the mechanisms underlying the decline of the immune function in the intestine. In that context, a dramatic decline in the density of mature M cells was observed in the Peyer's patches of aged mice [177], reducing the ability to transcytose particulate luminal antigens across the epithelium. Finally, the density of mononuclear phagocytes in Peyer's patches is not affected by aging [177] but the number and functionality of dendritic cells, which present antigens to immunocompetent B and T cells, are decreased what may explain the lack of oral tolerance in advanced age [178].

##### Endocrine function

Gastrin is a peptide hormone mainly secreted by the G cells of the stomach antrum, which stimulates postprandial acid secretion. Except in subjects suffering from *H. pylori* infection, plasma gastrin concentrations do not seem to be affected by aging [127]. An increase in gastrin receptor gene expression in the stomach has been reported in rodents [179] but similar studies in humans are not available. Secretin, which is secreted by S cells in the first part of the small intestine, stimulates bicarbonate pancreatic secretion, and inhibits gastric acid secretion. Whether aging affects this secretion is not known.

CCK is mainly released from I cells in the duodenum and ileum in response to the inflow of digesta into the small intestine. CCK stimulates the release of digestive enzymes from the pancreas and gallbladder contraction. Furthermore, this hormone increases intestinal motility, inhibits gastric emptying, and is considered as a strong anorexigenic gastrointestinal hormone. Using standard test meals, a lower CCK postprandial response has often been reported in elderly compared to young subjects [99, 180–182].

Ghrelin, an orexigenic hormone, is mainly produced in the stomach, but also in the proximal small intestine. In contrast to other gastrointestinal peptides, the synthesis of ghrelin increases with fasting. The effect of aging on total plasma ghrelin is unclear. Some studies reported a decreased basal ghrelin production in elderly [183, 184] but no differences were observed in other studies [99, 181, 185]. Also, the postprandial decrease in plasma ghrelin was either blunted [184, 185] or unaffected [99, 181]. Acyl-ghrelin is the active form of ghrelin. A recent study showed that, on the basis of a 24-h post-meal sampling, plasma acyl-ghrelin concentrations are lower in older adults than in young men [186].

PYY is primarily released from the ileum and the large intestine. In addition to inhibiting food intake, PYY also delays gastric emptying, inhibits intestinal motility, and decreases pancreatic secretions. Plasma PYY concentrations do not appear to be affected in the elderly [180]. In agreement with this finding, the density of the endocrine cells that produce PYY is not affected by aging [187].

The incretin hormones glucose-dependent insulinotropic polypeptide (GIP) and glucagon-like peptide 1 (GLP1) are produced at the beginning (K cells of the duodenum) and end (L cells of the ileum) of the small intestine, respectively, in response to the presence of nutrients within the digestive lumen. The main roles of GLP1 are to increase insulin secretion, decrease glucagon secretion from the pancreas, inhibit gastric acid secretion and gastric emptying, and decrease appetite and food intake. As GLP1, GIP promotes insulin secretion by the pancreas. Although a majority of studies showed no significant alteration in postprandial GLP1 and GIP response in non-diabetic elderly [99, 180, 188], one study reported an increased postprandial secretion of GIP and GLP1 in postmenopausal women [189]. Glucagon-like peptide 2 (GLP2) is co-secreted with GLP1 by the L-cells in response to the ingestion of nutrients. GLP2 stimulates intestinal growth and mucosa repair, improves nutrient absorption, and slows down gastric motility. The effect of aging on GLP2 secretion is not known.

In conclusion, the main changes in the release of gastrointestinal peptides in advanced aged are an alteration of the postprandial response of CCK and ghrelin.

### Consequences of the aging GIT on nutrient bioavailability

#### Macronutrients

Although the secretion of pepsin and pancreatic enzymes declines with advancing age (see Section 2.1.3), proteolytic activity in the small intestine still appears to be sufficient to ensure a proper digestion of proteins in the elderly. However, studies in aged rodents showed a lack of adaptability of protein digestion to nutritional stress, such as food restriction [190] or the presence of antinutritional factors in the diet [191]. Because of their technical difficulty, measurements of protein digestibility in the small intestine of humans are scarce and none has been performed in the elderly. It is, thus, not known if brush-border peptidases, peptide transporters, and amino acid transporters are affected by aging. Nonetheless, scientific evidence suggests that these proteins are not limiting the absorption of amino acids in the small intestine of the elderly. However, peripheral availability of amino acids could be strongly affected by an increased metabolic use of dietary amino acids in the GIT and the liver [192, 193].

As for protein digestion, it is not known if starch and lipid digestion in the small intestine are adversely affected by the small decline in amylase and lipase secretion observed in the elderly. Indeed, no data is available in the literature on the digestibility of starch or lipid in the small intestine of elderly. More specifically, it is not known whether the bioavailability of essential fatty acids (EFAs), in particular linoleic acid (LA, 18:2 n-6) and α-linolenic acid (ALA, 18:3 n-3) is impaired with advancing age, although one study reported the same bioavailability of ALA in young subjects and 45–69 y subjects [194].

#### Vitamins and minerals

Vitamin B12 deficiency concerns about 15% of the elderly population [195]. This deficiency can be due to either malabsorption of food-bound cobalamin or to an insufficient dietary intake, the latter mostly resulting from a decrease intake in animal products. Food-bound vitamin B12 is released by pepsin in the acidic environment in the stomach where it binds to a gastric protein (the R binder). Subsequently, vitamin B12 is released by pancreatic enzymes in the small intestine where it binds with intrinsic factor. The cobalamin-intrinsic factor complex then binds to the ileal endocytic cubam receptor formed of two proteins, cubulin and amnionless. The cubam receptor mediates endocytosis of the intrinsic factor-cobalamin complex, which is then degraded in lysosomes to release cobalamin into plasma in complex with transcobalamin II. Cobalamin malabsorption in the elderly could therefore stem from a decrease in pepsin or acid secretion, a lack of intrinsic factor, or other defects in the cobalamin uptake system [196].

The digestion and absorption of the fat-soluble vitamins A, D, and E basically follow the same path as lipids. As such, membrane proteins are involved in the absorption of these vitamins [197–199]. The effect of aging on the expression of these carriers and on the bioavailability of fat-soluble vitamins is not known.

Intestinal absorption of calcium decreases with age [200, 201]. Calcium absorption has both an active (1,25(OH)_2_D_3_-dependent) and passive component (see Section 2.1.4.1). Elderly women have an impaired intestinal response to 1,25(OH)_2_D_3_ that may contribute to their negative calcium balance and bone loss [202]. The dietary source of calcium (milk, calcium carbonate, or fortified orange juice) does not seem to affect its relative bioavailability in elderly [203]. The effect of aging on the bioavailability of the other minerals is not documented. However, for some of these minerals, such as iron, the luminal pH is an important factor for their absorption. As such, the hypochlorydria observed in elderly subjects suffering from atrophic gastritis could decrease the bioavailability of these minerals [204].

#### Water

The GIT is the location of very intense water fluxes. On a daily basis we consume about 2 L of water and about 7 L of water are secreted into the GIT via the different digestive secretions. The largest part of this water is reabsorbed in the small intestine (about 8 L) and, to a lesser extent, by the colon. The high permeability of the small intestine ensures a rapid osmotic rebalancing of the digestive content during the absorption of ions and nutrients. Water can cross the GIT epithelium via the paracellular or transcellular route. The relative significance of both routes in the different digestive organs has been reviewed elsewhere [205]. The transcellular route involves three different mechanisms, namely passive diffusion, cotransport with ions and nutrients (for example through SGLT1 transporter), and the water channels named aquaporins [206]. Currently it is not known if potential modifications in tight junction proteins, membrane transporters, or aquaporins can impaired water bioavailability in the elderly.

## CONSEQUENCES OF MALNUTRITION ON FUNCTIONAL DECLINE OF ORGAN SYSTEMS

Malnutrition and aging are associated with progressive deterioration of health and physical performance in older adults. This deterioration leads to decreased functional abilities, dependency in activities of daily living, poor quality of life, and further decline in physical activities.

As can be concluded from Part 2 of this review, age-related changes in the gut may enhance the occurrence and severity of malnutrition by several paths that differ by the specific GIT organ system. In this part, the consequences of malnutrition on the most sensible organ systems of the elderly will be addressed. The impact of malnutrition on the severity of diseases, health complications and mortality is also presented.

### Nervous system

Altered cognitive functions and neurodegenerative diseases may be the cause or the result of malnutrition. Likewise depression may either be the cause or the result of decreased dietary intake as specific deficiencies in nutrients may accelerate depressive symptoms [207]. Indeed, depressive symptoms are more prevalent in individuals with impaired nutritional status manifested in diets of poor quality, unintentional weight loss, and a decreased intake of specific nutrients [208, 209]. In particular, deficiencies in vitamin B9 (folate) [210], vitamin B12 [211], vitamin B6 [212], and polyunsaturated fatty acids (PUFAs) are associated with depressive symptoms [213] and declining cognitive functions [214] in older age.

The brain is a site of high metabolic activity and is especially prone to oxidative stress and damage to neural tissue [214]. A prevailing theory is that oxidative damage and neural inflammation are the underlying biological mechanisms of neurodegenerative disorders like Alzheimer's disease and Parkinson's disease [215, 216].

Oxidative stress, which is defined as an excess of reactive oxygen species (ROS), is also the main mechanism inducing damage to the retina in age-related macular degeneration (AMD). Oxygen exacerbates physiological and molecular damage to the eye during aging via free-radical chain reactions [217]. The relation of ROS to diet is two-edged as the metabolism of macronutrients enhances the production of ROS and some nutrients can inhibit this process.

ROS appears to play a major role also in the degeneration of hearing during aging [218, 219]. Age-related hearing loss, or presbycusis, is a complex degenerative disease and one of the most prevalent chronic conditions in elderly, affecting tens of millions worldwide [220]. As for other neurodegenerative diseases, hearing loss is inversely associated with the intake of antioxidant vitamins, although most studies were performed in animal models. In particular, rats and dogs fed a diet rich in antioxidant vitamins showed less degeneration of the spiral ganglional cells and the stria vascularis compared to animals fed a control diet [221, 222].

#### Antioxidant vitamins and phytochemicals

The nutrients involved in the counteraction of oxidative damage include elements such as Mn, Cu, and Se, which are part or co-factors of antioxidant enzymes, as well as the antioxidants vitamin E and vitamin C. Carotenoids and flavonoids may also be indirectly involved in cellular mechanisms protecting against oxidative stress. The antioxidant nutrients may play more important roles in the aging brain than in other organs of the body because of the reduction in the number of antioxidant enzymes that provide neuronal protection [223]. A reduced intake of antioxidant nutrients, which is characteristic of diets of poor quality and of malnutrition, may thus adversely affect cognitive function.

There is a weak evidence to support protection against dementia by dietary intake of nutrients such as vitamin C and β-carotene. The single dietary antioxidant with a prominent evidence for a protective effect on cognition is vitamin E. Indeed, prospective epidemiological studies on dietary vitamin E consistently showed statistically significant inverse associations with incident dementia, Alzheimer's disease, and cognitive decline [223, 224].

Molecules with an effect on AMD susceptibility include carotenoids (lutein, zeaxanthin, β-carotene), which show the most convincing results, although still inconsistent. In addition, weak protective effects of vitamins (A, B, C, D, E), minerals, dietary fats, and dietary carbohydrates were also suggested [225].

As mentioned above, studies on the relationships between lutein, zeaxanthin, β-carotene and AMD are conflicting. A case-control study focusing on the effect of carotenoids on AMD was performed by the Eye Disease Case-Control Study Group [226]. This study recruited 421 patients with neovascular AMD and 615 controls. High serum levels of carotenoids were associated with a reduced risk of neovascular AMD. In particular, the odds ratios for AMD in subjects with sufficient intake of lutein, zeaxanthin, β-carotene, alpha-carotene, and cryptoxanthin ranged from 0.3 to 0.5. However, overall consistency across studies was lacking. Some studies failed to identify a correlation between AMD and the intake of vegetables, antioxidant vitamins, or carotenoids. Other studies reported a direct inverse association between the dietary intake of lutein and zeaxanthin and the occurrence of AMD [227, 228]. Notably, a large study of 4,519 participants, performed by the Age-Related Eye Disease Study Research (AREDS) Group, reported a low likelihood of AMD in people with a high dietary intake of lutein and zeaxanthin [226]. Carotenoids intake is related to the intake of fruits and vegetable. In that regard, the National Health and Nutrition Examination Survey showed an inverse association between the frequency of intake of fruits and vegetables rich in carotenoids and the prevalence of AMD [229].

A review by Zampatti et al. [225] that includes several studies reported a weak or non-existent association between serum vitamin E levels, the consumption of vitamin E supplements, and the risk of AMD. The AREDS Group demonstrated an inverse association between the intake of vitamin C and E and neovascular AMD [226]. However, these results were not repeated in other studies. In particular, no significant association between vitamin C intake and AMD was observed in the Eye Disease Case-Control Study Group or in the POLA study [226, 230]. The National Health and Nutrition Examination Survey study showed an inverse association between the plasma levels of vitamin D and early AMD, whereas an association was no longer reported for advanced AMD [231]. Of note, the anti-inflammatory properties of vitamin D are of additional interest in the prevention of AMD in light of the inflammatory component of AMD [232].

AMD weakly and inversely associates with the intake of minerals such as zinc, copper, and selenium. These minerals are at risk for deficiency in the general population with increased incidence among the elderly. The food sources for these minerals are specific and costly, including red meat and poultry (as sources of zinc) as well as bread, grain, meat, fish, and eggs (as sources of selenium). The intake of meat, poultry, and fish is, thus, often compromised in the elderly, leading to dietary deficiencies and malnutrition.

Finally, the Korea National Health and Nutrition Examination Survey conducted a study in South Korea demonstrating that dietary intake of vitamin C was positively associated with hearing quality in an elderly cohort. However, due to high rates (> 50%) of insufficient intake of vitamins in the control group, a proper diet in itself may have prevented hearing decline [218].

#### B vitamins

Among the B vitamins, vitamin B9 and B12 have received the greatest attention for brain health in the scientific literature. These vitamins are co-factor nutrients that modulate neurocognitive development and neurodegeneration. Data from epidemiological studies and randomized clinical trials on the relationship between vitamins B9 and B12 and cognitive deterioration is conflicting [233] and depends on the study designs and the various methods used for evaluating cognitive function. In recent years, vitamins B9 and B12 have received a lot of attention as risk factors for dementia. This interest was largely based on their function as co-factors in the metabolism of homocysteine. Homocysteine has been associated with the risk of developing Alzheimer's disease in some [234, 235] but not all studies [236]. The role of homocysteine is thus still debated, even though its mechanism of action in this pathology is not known. Of note, homocysteine deficiency is neurotoxic in mouse models of Alzheimer's disease [237].

Elderly at nutritional risk are characterized by low levels of vitamin B12. Even among elderly with appropriate intake, blood levels of vitamin B12 are frequently low [233, 238, 239]. The intake of B vitamins, particularly vitamin B12, folic acid, and vitamin B6 are related to homocysteine levels. As AMD is also associated with elevated levels of plasma homocysteine, B vitamins are of particular interest in the prevention of AMD. Indeed, plasma vitamin B12 concentrations were lower in patients with exudative AMD compared to controls and patients with AMD [240].

Among women participating in the Health, Aging, and Body Composition (Health ABC) Study [241, 242], a poor vitamin B12 and folate status was associated with age-related auditory dysfunctions. In particular, women with an impaired hearing had lower serum vitamin B12 levels (38%) and lower red cell folate levels (31%) than women with normal hearing. These results were confirmed in women taking supplements of B12 or folic acid.

#### Polyunsaturated fatty acids

The EFAs LA and ALA, and their longer and more unsaturated derivatives, arachidonic acid (ARA, 20:4 n-6), eicosapentaenoic acid (EPA, 20:5 n-3), and docosahexaenoic acid (DHA, 22:6 n-3), play key roles in both cell structure and function, and are thus indispensable for brain development. ARA and DHA are found in large concentrations in brain lipids, and nearly 6% of the dry weight of the brain is built of n-3 PUFAs [243]. PUFAs are incorporated into phospholipids and are key components of the brain neuronal and glial cell membranes. PUFAs provide fluidity and the proper environment for an active functioning of integral proteins. Moreover, PUFAs esterified in phospholipids have a role in cellular function because they are a reservoir of signaling messengers for neurotransmitters and growth factors. PUFAs regulate both eicosanoid and proinflammatory cytokine production, which play a key role in depression and neurodegenerative diseases linked to aging. Overall, n-3 PUFAs are considered anti-inflammatory while n-6 PUFAs pro-inflammatory. In addition, PUFAs are involved in the regulation of gene expression. n-3 and n-6 PUFAs compete for enzymes involved in both biosynthesis of long-chain PUFAs (LC-PUFAs) from the corresponding EFA and conversion of specific LC-PUFAs to eicosanoids. Considering that LA conversion is more efficient than ALA conversion, an excess of the n-6 precursor LA promotes the formation of ARA and related pro-inflammatory eicosanoids [244].

LC-PUFAs are not only synthesized from EFAs, but can also be introduced with food. DHA and EPA are mainly found in fish oils and fatty fishes whereas ALA is commonly found in vegetable oils. The dietary intake of n-6 PUFAs (both as LA and LC-PUFAs) largely exceeds the intake of n-3 PUFAs. PUFAs are believed to have occurred in early diets at a ratio n-3:n-6 of 1:1. Nowadays, this ratio ranges between 10:1 and 30:1 and this dietary imbalance may increase susceptibility to neuronal damage.

Experimental studies have shown that n-3 PUFAs are involved in many neurobiological processes, indicating that they may prevent age-related brain damage [245].

### Muscoskeletal system

#### Age-related changes in body composition and malnutrition

In healthy adults, muscles constitute over half of total body protein. The muscle mass, however, decreases with age due to greater rates of protein breakdown than synthesis. Aging is also accompanied by an infiltration of adipose cells in muscle tissues, which is associated with a decreased muscle strength and insulin resistance. Changes in ‘muscle quality’, expressed as the amount of force produced per unit of muscle mass, explain much of the strength loss during aging. These age-related neuromuscular modifications include reductions in the number, size, and contractility of muscle fibers, as well as skeletal muscle fat infiltration. Neuromuscular changes along with increased body fat, systemic low-level inflammation, and oxidative stress contribute to further deteriorations of the muscoskeletal system eventually leading to sarcopenia [246], osteoporosis, weight loss, and frailty.

Sarcopenia was defined by the European Working Group on Sarcopenia in Older Persons (EWGSOP) as a loss of muscle mass in combination with a loss of muscle strength or physical performance [247]. Sarcopenia is measured with a range of indicators, including relative skeletal muscle mass, total muscle mass, handgrip, and physical performance, and affects up to one-quarter of older adults [247]. Sarcopenia is associated with a reduced ability to complete everyday tasks and an increased risk of falls, this phenomena resulting in a loss of independence in elderly [248].

The decline of bone mineral density (BMD) with increasing age can lead to osteopenia and, in extreme cases, to osteoporosis. The latter is a significant health problem that contributes to disability and premature mortality among older women and men. Although genetic factors influence peak bone mass, diet is clearly one of the modifiable risk factors for osteoporosis [249]. Elderly are generally advised to strengthen their skeletal health by following a nutrient-dense diet that is also diverse and rich in fruits and vegetables. This diet should contain adequate amounts of proteins, vitamins (B, C, D, K), minerals (calcium, potassium, magnesium), and trace elements [250, 251].

Frailty is a geriatric syndrome characterized by a reduction of the physiological functional reserves and a decreased homeostatic capacity leading to greater vulnerability to adverse health outcomes including falls and fractures and increased risk of death. About 30% of community-dwelling persons older than 65 y experience one or more falls every year. About 5% of falls result in a fracture and more than 90% of the hip fractures are attributable to falls. The consequences of hip fractures are severe: 50% of older persons have permanent functional disabilities, 15% to 25% require long-term nursing home care, and 10% to 20% die within one year [252]. The decrease in food intake associated with anorexia leads to a reduction in exercise capacity and to a decline in muscle mass and strength, and is therefore involved in the development of the frailty syndrome.

Sarcopenia, osteoporosis, and frailty are worsened by malnutrition and specific nutrients deficiencies, in particular protein, antioxidant vitamins, minerals, and fatty acids.

#### Proteins

The National Health and Nutrition Examination Survey (NHANES) revealed that approximately one-third of adults over 50 y of age fail to meet the Recommended Dietary Allowance (RDA) for protein, while approximately 10% of older women fail to meet even the Estimated Average Requirement (EAR) (0.66 g protein/kg/day) [253]. A short fall of protein supplies relative to the needs can lead to loss of lean body mass, particularly muscle loss. The association between dietary protein intake, using a food frequency questionnaire (FFQ), and lean body mass, measured by dual energy x-ray absorptiometry (DEXA) over a 3 y period, was assessed in the Health ABC Study. After adjustment for potential confounders, participants in the highest quintile of protein intake lost approximately 40% less lean body mass than those in the lowest quintile of protein intake [254].

To maintain and regain lean body mass and muscle function, the PROT-AGE Study Group [255] recommended an average daily intake of at least 1.0–1.2 g protein per kilogram of body weight. Furthermore, increasing dietary protein should be integrated into an energy-controlled dietary plan for weight management. Dietary enrichment with leucine or a mixture of branched-chain amino acids (BCAA) may also help enhancing muscle mass and function [256]. β-hydroxy-β-methylbutyrate, a bioactive metabolite derived from leucine, showed some positive effects on muscle mass and function in some studies. The sample size of these studies was, however, small [247].

Several epidemiological studies reported that protein intake correlates positively with BMD and negatively with the rate of bone loss [255]. A prospective cohort study showed that elderly subjects with osteoporosis had a higher BMD when their daily protein intake exceeded 0.8 g/kg body weight or comprised at least 24% of their total energy intake [257]. This finding was confirmed by a systematic review of this research field [258].

#### Antioxidants, vitamins, and phytochemicals

In Canadian adults aged 60–75 y, the odds for sarcopenia were greater in those who reported failing to meet the RDA for the antioxidants selenium and vitamins A, C, and E [259]. The Hertfordshire Cohort Study also observed a positive association between handgrip strength and the intake of β-carotene, selenium, and vitamin C [260]. The Women's Health and Aging Study (WHAS) recruited nearly 700 community-dwelling women aged 70–79 y and reported an inverse correlation between plasma carotenoid and vitamin E concentrations and the odds for low muscle strength and frailty [261]. Over 3 years in this study, low baseline plasma concentrations of carotenoids were associated with a decline in walking speed [262]. Finally, low plasma concentrations of selenium were also associated with poor muscle strength in the CHIANTI Study [210].

Oxidative stress is a major mechanism in the loss of bone mass and strength as ROS influence the generation and survival of osteoclasts and osteocytes. In this context, dietary antioxidants such as vitamins (A, E, C) and phytochemicals such as carotenoids and flavonoids (quercetin) may play a protective role in bone health of elderly [250, 251].

Vitamin D plays an important role in bone and mineral metabolism, and its deficiency is closely associated with metabolic bone disease. Vitamin D is a secosteroid hormone produced by the skin following its exposure to ultraviolet B light. Vitamin D is also obtained from the diet, albeit in small amounts. Although controversy surrounds the definition of low vitamin D status, there is a general acceptance that the optimal circulating 25-hydroxyvitamin D_3_ (25(OH)D_3_) level should be at least 75 nmol/L. A threshold for optimal 25(OH)D_3_ and hip BMD has been established from 13,432 individuals in the Third National Health and Nutrition Examination Survey (NHANES III). NHANES III included both younger (20–49 y) and older (≥ 50 y) individuals with different ethnic background. This study shows that serum 25(OH)D_3_ levels were positively associated with BMD throughout the reference range of 22.5–94 nmol/L in all subgroups [263].

Vitamin D deficiency is recognized as a worldwide epidemic, especially in the elderly, as a result of decreased sun exposure and, consequently, decreased intrinsic synthesis, lower dietary intake, and decreased vitamin D receptor (VDR) activity. More than 60% of postmenopausal women have deficient 25(OH)D_3_ serum levels, including populations in sunny countries such as in the Middle East, in Australia and in Spain. In elderly, vitamin D insufficiency and deficiency appear to be particularly prevalent in nursing home residents. Inadequate levels of vitamin D lead to a reduced intestinal calcium absorption, secondary hyperparathyroidism, impaired mineralization, and increased bone resorption [264]. The Institute of Medicine consequently recommends a daily dose of vitamin D supplement (800 IU) for elderly over 71 y [265].

Vitamin D affects fracture risk through its effects on bone metabolism and, consequently, on the risk of falling. Daily supplementation of 800 IU vitamin D is needed to positively influence the rate of falls [266]. A meta- analysis published in 2005 showed that oral supplementation of 700–800 IU vitamin D reduces the risk of hip and non-vertebral fractures in ambulatory or institutionalized elderly [267]. Brincata et al. recommended in 2015 that a deficiency in vitamin D should be aggressively treated with higher pharmacological doses to achieve serum levels ≥ 75 nmol/L [264].

Nuclear VDRs in skeletal muscles bind 1,25(OH)_2_D_3_, the active form of vitamin D, and promote protein synthesis. The apparent decrease in VDRs within the muscle during aging may explain part of the age-related decline in protein synthesis [268]. Prospective studies suggested that vitamin D may be important for muscle function. The majority of studies in the research field of sarcopenia assesses 25(OH)D_3_, a precursor of 1,25(OH)_2_D_3_. In the Longitudinal Aging Study Amsterdam (LASA) the odds of losing more than 3% of muscle mass over 3 y were close to two times greater in participants with low plasma levels of 25(OH)D_3_ at baseline (< 25 nmol/L) compared to participants with high levels (> 50 nmol/L) [269]. In the same study, the odds for a loss of grip strength greater than 40% over 3 y were around two times greater for participants with low serum 25(OH)D_3_ levels at baseline than for those with high levels. Vitamin D may, therefore, play an important role in the maintenance of muscle function in elderly. In conclusion, plasma levels of 25(OH)D_3_ should be measured in elderly with muscle loss and values lower than 75 nmol/L should trigger dietary interventions and/or supplementation [269].

#### Calcium

Calcium is a key architectural component of bones and, thus, critical for the maintenance of bone health. Inadequate calcium absorption increases the concentration of parathyroid hormone leading to increased bone resorption. In the USA the RDA for calcium was recently raised to 1,200 mg/d for females aged 50–70 y [265]. For males, the recommendation remained at 1,000 mg/d whereas it was set at 1,200 mg/d for both genders over 71 y. The calcium intake of postmenopausal women in Europe typically falls well below recommended values [270]. More than 50% of the older NHANES participants in 2005–2006 (a representative national survey of older adults in USA) failed to achieve the RDA for calcium, even with supplements. Furthermore, 1,384 members of the NHANES cohort aged 50–70 y and 71 + y, were tested for the association between total calcium intake and hip and spine BMD after adjusting for BMI. The total calcium intake ranged from a mean of 400 mg/d in the first quintile to 2,100 in the fifth quintile. However, little difference in hip or lumbar BMD was found in relation to total calcium consumption in women and men across the five quintiles. In particular, a dietary intake of calcium approaching or even meeting the current recommendations was not related to higher BMD of the hip or lumbar spine compared to the elderly subjects with lower intakes of calcium [271].

#### Polyunsaturated fatty acids

Several studies suggest a potential role of fatty acids on muscle, particularly n–3 LC-PUFAs, which predominately consist of EPA and DHA. The Hertfordshire Study, a large retrospective cohort study of nearly 3,000 community-dwelling older men and women aged 59–73 y, found a positive association between fatty fish consumption and grip strength [260]. The Reykjavik Study, a prospective cross-sectional study with a cohort of subjects aged 66–96 y at baseline, followed over 5 y the association between plasma PUFAs and measures of muscle size, intermuscular adipose tissue, and muscle strength. The results revealed inconsistent cross-sectional relations between the various plasma PUFAs measured and indicators of muscle health, including muscle size and strength, and intermuscular adipose tissue [272].

In pigs, maintaining dietary n-6:n-3 PUFA ratios of 1:1–5:1 facilitates the absorption and utilization of fatty acids and free amino acids, and results in improved muscle and adipose composition [273].

The effects of n-3 PUFAs on BMD are unclear. Three studies found a positive relation between the consumption of n-3 PUFAs and BMD (total body, spine) [249, 274, 275]. In a cohort of 1,417 Chinese elderly living in the community fish intake was negatively associated with bone loss in hip and femoral neck, albeit only in men. A recent meta-analysis, however, concluded that the data currently available is insufficient to conclude on a positive effect of n-3 PUFAs on bone health [276].

### Cardiovascular system

#### Cardiovascular disease

In general, the contribution of malnutrition to cardiac illness has been underestimated. Conventional medical theories relate cardiac disease and atherosclerosis to overnutrition, rather than undernutrition. Nonetheless it should be remembered that the heart is a muscle and thus becomes vulnerable to numerous micronutrient deficiencies such as vitamin A, vitamin C, vitamin E, thiamine, B vitamins, vitamin D, selenium, zinc, and copper [277].

These deficiencies compromise heart muscle function and may lead to heart failure. Additionally, the poor cardiac output emanating from prior cardiac injury (*e.g*. myocardial infarction) may lead to intestinal edema secondary to malabsorption and cardiac cachexia, an unintentional severe weight loss caused by heart disease [278]. Obviously these would all hasten complications and death. For example, in a study involving 171 patients with heart failure, cardiac cachexia was predictive of 18-month mortality independent of age, symptomatic classification of heart diseases according to the New York Heart Association (NYHA), left ventricular ejection fraction, and exercise tolerance [279]. Another relevant factor is that cardiac patients tend to restrict their diet sometimes excessively as part of genuine or perceived recommendations for highly restrictive diets. Total adherence to such diets induces a vicious cycle characterized by malnutrition and deficiencies in the above mentioned nutrients, which ultimately aggravates cardiac failure and leads to clinical deterioration. Interestingly, Lennie et al. [280] proposed that factors such as decreased hunger sensation, diet restrictions, fatigue, shortness of breath, nausea, anxiety, and sadness may contribute to a decreased nutritional intake in patients with heart failure. Additional mechanisms for a poor micronutrient status in patients with heart failure include a decreased intestinal absorption as a consequence of gut edema, increased urinary losses due to drug therapy [212], and increased oxidative stress.

In conclusion, although malnutrition is secondary to cardiometabolic diseases, particularly heart failure, micronutrients deficiencies appear to significantly increase the morbidity and mortality associated with cardiovascular diseases (CVD).

#### Anemia

The prevalence of anemia increases with age and is a common condition in the elderly. Anemia affects the quality of life through cognitive and physical functions. It is a comorbid condition that affects other diseases (*e.g*. heart disease, cerebrovascular disorders) and is positively associated with the risk of death [281, 282]. Next to anemia mediated by inflammatory processes, iron deficiency anemia is the second most common cause of anemia in the elderly [283]. Iron deficiency anemia usually results from chronic gastrointestinal blood loss. In addition, malnourished elderly are at increased risk for iron deficiency due to inadequate dietary intake, absorption, and bioavailability of iron. This phenomena is worsened by the intake of anti-acid drugs [284]. As mentioned earlier in this review, both vitamin B12 and folate deficiency are common among elderly, each occurring in at least 5% of anemic patients [285]. In the elderly, folate deficiency usually develops as a result of inadequate dietary intake [281]. In the case of vitamin B12, intestinal malabsorption of cobalamin results from changes in the functionality of the GIT (see Section 2.2.2), explaining over 50% of vitamin B12 deficiencies, the remaining being attributed to a poor dietary intake [233].

### Immune system

The classical view of immune aging is of an immunodeficiency state that predisposes to progressive T-cell dysfunction with advancing age. Immunosenescence reflects the decreasing ability of the aging organism to react to the attacks of external agents such as pathogens by mounting an efficient immune response. It is generally characterized by a decreased proliferation of T lymphocytes and an impaired T-helper activity, which lead to impaired cell-mediated and humoral responses to T cell-dependent antigens. Morely et al. suggested that an insufficient intake of energy and protein distorts different components of the immune system, as reflected by a delayed cutaneous hypersensitivity and decreased total lymphocyte count, T-cell proliferation, and interleukins [207, 286]. In addition to the well-recognized phenomenon of immunesenescence, aging is also characterized by an increase in systemic low-grade inflammation (“inflammaging”), which predisposes elderly to the development of chronic inflammatory diseases [287]. In summary, whereas the innate immune of elderly is maintained if not enhanced, aging of the immune system is mostly characterized by a decline in acquired immunity [288].

In addition to the pathogen load, the nutritional status is a major factor influencing T cell responses [288]. Evidence is accumulating suggesting that a suboptimal status of essential nutrients, as is the case in malnutrition, contributes to the immunological defects observed with aging. In addition to dietary lipids, the main nutrients involved in immune function are water-soluble vitamins (B6, folate, B12, C). Among the fat-soluble vitamins, the most convincing evidence was obtained for vitamins A, D, and E. These nutrients selectively influence the immune response and support a coordinated host response to infections. Deficiency may thus impact virulence of otherwise harmless pathogens [289]. For illustration, a study reported that 200 IU of vitamin E supplementation administered to nursing home residents significantly reduced the risk of upper respiratory tract infection [290]. Another micronutrient receiving increased attention for its role in host defenses is vitamin D in particular as Toll-like receptor-mediated mechanisms appear to be most vulnerable to subclinical vitamin D deficiency [291]. Therefore, vitamin D may have particular relevance to frail older adults with limited exposure to the sun and who do not receive supplementation. Of note, an increased intake of some of these nutrients above the recommended levels even appears to be needed to maintain a proper function of the immune system and to reduce the incidence of infections in the elderly [288, 292].

Trace elements found in the diet and involved in immune function, such as selenium, zinc, copper, and iron, should also be considered. For illustration, a recent study involving nursing home residents found that subjects with low zinc concentrations had a lower incidence of pneumonia, a shorter duration of pneumonia, and fewer days of antibiotic use than did those with normal zinc serum concentrations [293].

The immune system is influenced by dietary lipids that are precursors of eicosanoids, prostaglandins, and leukotrienes. Lipids may thus also have important immunomodulatory effects, especially PUFAs, which regulate the inflammatory process and the immune response [294]. As discussed earlier in Section 3.1 devoted to the nervous system, an increased intake of n-3 PUFAs is recommended to prevent and ameliorate inflammatory and autoimmune diseases as well as several age-related diseases in which inflammation plays a role. However, as for pharmacology, the complex nature of immunity cautions against a general “one-fits-all” recommendation and a proper balance between the immune response and inflammation must be achieved, even for the identification of dietary solutions.

Malnutrition in the elderly is not only associated with a diminished immune response to pathogens but also with an impaired response to vaccines. In elderly, especially those in nursing homes, malnutrition indeed appears to impair vaccine efficacy due to an adequate immune response [295]. Three vaccines including influenza vaccine, pneumococcal polysaccharide vaccine, and herpes zoster vaccine, are specifically recommended by the Centers for Disease Control and Prevention for adults over 65 y of age [296].

### Skin system

Nutrient deficiencies or malnutrition can have negative effects on wound healing by prolonging the inflammatory phase, decreasing fibroblast proliferation, and altering collagen synthesis [297]. Malnutrition has also been related to a decreased wound tensile strength and increased infection rates [298]. Malnourished patients can develop pressure ulcers and infections that delay wound healing and result in chronic non-healing wounds. Chronic wounds represent a significant cause of morbidity and mortality for many elderly patients.

Malnutrition increases the risk for the occurrence of pressure sores. Low protein and energy intake, a low BMI, and albuminemia are risk factors for the development of pressure sores in the elderly [299]. A meta-analysis of four clinical studies showed that oral nutritional supplements could significantly reduce the incidence of pressure ulcer development in at-risk patients with an odds ratio of 0.75 [300]. On the other hand, data on the effect of the nutritional status of elderly on the healing of existing pressure ulcers is scarce and not entirely convincing. However, the existing data suggests that malnutrition slows down the healing process and that an increase in protein and energy intake increases the rate of healing. Overall, the consensus is that nutrition is important for wound healing [300].

### Well-being, overall health, and mortality

The impact of malnutrition on the well-being of elderly has not been studied systematically until recently. A pooled analysis of fifteen studies reporting an association between the nutritional status of elderly and their quality of life concluded that the overall (physical) quality of life was almost threefold better in well-nourished participants compared to individuals with malnutrition (OR: 2.85; 95% CI: 2.20–3.70) [301].

Numerous studies in acute-care hospital settings have demonstrated a strong inverse association between specific clinical markers of the protein-energy nutritional status and the risk of morbidity and mortality [45, 302]. Other studies examined the association between the nutritional status and clinical outcomes during hospitalization or after hospital discharge [303, 304]. Malnourished elderly and elderly at nutritional risk tend to have longer hospital stays, delayed wound healing, higher incidence of complications, higher re-admission rates, and higher mortality rates, when compared to their well-nourished counterparts [45, 305]. For illustration, severely malnourished hospitalized elderly patients were more likely than well-nourished patients to be dependent in their daily activities three months after discharge. They were also more likely to spend time in a nursing home during the first year after discharge [299, 303]. In 185 elderly hospitalized patients, a low energy intake was an independent risk factor for nosocomial infections [15, 306, 307]. Finally, episodes of sepsis occurred significantly more often in severely undernourished hospitalized elderly patients [308].

An association between mortality and malnutrition or nutritional risk has been consistently reported in elderly subjects as assessed by BMI, weight loss, plasma levels of albumin, and food intake [45, 309]. Several prospective studies performed in the community showed that weight loss and a lower dietary intake, as measures of nutritional risk, were associated with increased mortality, independently of the health status. One study reported an increased risk (RR = 2) for mortality when comparing weight losers and elderly with a stable weight [310]. Another study with 288 elderly showed that weight loss was a significant predictor of mortality with a RR of 1.76 [304]. Other indicators of malnutrition or nutritional risk, including serum albumin and variables related to inflammation, showed a mortality RR of 2.3. A significant RR for mortality of 3.7 was also measured among elderly women with low serum albumin levels, a significant RR of 1.9 being observed for men [299]. In hospital settings, mortality doubled in the most underweight elderly (BMI < 18 kg/m^2^) compared to subjects with a BMI ranging between 20 and 40 kg/m^2^. In patients aged 70–79 y, mortality even tripled in the patients with BMI < 18 kg/m^2^ compared to the patients with a BMI ranging between 32 and 40 kg/m^2^ [308]. This data suggests that aging increases the risk of mortality in severely underweighted patients. In addition, weight loss was the best predictor of mortality one year after admission and hospital discharge [311]. During acute hospitalization both age and albumin were significant predictors of mortality, low albumin levels on admission being associated with a relative risk of death of 3.7 [312]. Finally, a group of elderly with low nutrient intake had a dramatically higher rate of in-hospital mortality (RR = 8.0) and 90-day mortality (RR = 2.9) compared to the control [302]. Taken together, an inverse correlation between the quality of the nutritional status and mortality is undisputed.

## DIETARY SOLUTIONS FOR THE AGING GASTROINTESTINAL TRACT

### Factors influencing nutrient requirements and food intake in elderly

The nutritional status in elderly is multi-factorial and is both influencing and influenced by the health status. A balanced diet with all the specific nutrient requirements, a physically active healthy lifestyle, avoidance of tobacco, and maintaining a healthy body weight are key factors to prevent malnutrition and chronic diseases and keep older adults healthy, independent, and community dwelling [313]. When a risk of malnutrition is diagnosed, it is essential to combine a nutritional support with measures for the identification and correction of risk factors [314]. Health-promoting interventions implemented individually, such as exercise programs, preventive home visits, comprehensive geriatric evaluation and management, and attention to adequate nutrition with or without nutritional supplements, have been shown in separate studies to be both feasible and effective in reducing age-related deterioration [315].

The complex set of functional changes taking place along the GIT, described in Part 2 of this review, which preterits an efficient distribution of nutrients to the organism of elderly, and the dramatic impact of malnutrition on the health status of elderly, highlighted in Part 3, underline that dietary solutions aimed at preventing and treating malnutrition in elderly must be developed based on an understanding of the factors influencing nutrient requirement, food intake, and the capacity of the senescent GIT to extract nutrients from the food matrix and to absorb them.

### Dietary guidelines against malnutrition in the elderly

General dietary recommendations for older adults are addressed in Dietary Reference Intakes (DRIs) in the USA [316], considering age categories 51 y to 70 y and > 70 y. The European Food Safety Authority (EFSA) established Dietary Reference Values (DRV) for total carbohydrates and dietary fiber [317], fats [318], water [319], and protein [320] for the European population. In 2013, the EFSA reviewed energy recommendations, considering age categories of 60–69 y and 70–79 y [321]. Revision of DRVs for micronutrients is ongoing [322] but the existing recommendations do not yet consider specific amounts for older adults. The current guidelines are thus merely indicators since actual nutrient requirements depend on all the factors listed on the previous sections.

More specific recommendations to prevent and deal with malnutrition in elderly have been addressed by several health authorities and reviews. To reduce risk of protein-energy wasting and frailty in Europe, a daily intake of 30–40 kcal/kg and 1.0–1.5 g protein/kg body weight was proposed, whereas the exact recommended values depend on the health status [314, 323, 324]. In the USA, the Academy of Nutrition and Dietetics suggested a range of daily protein intake between 1.0 and 1.6 g/kg, with a consumption of 25–30 g of high quality protein at each meal [313]. Protein pulse feeding and the consumption of fast proteins (such as whey protein) may promote muscle protein synthesis and prevent sarcopenia in older adults [323, 325].

Several reviews described that older adults are at higher risk of vitamin and mineral deficiencies, although their energy intake is within recommendations, particularly vitamins A, B1, B2, B12, D, E, K, calcium, and potassium [313, 326, 327]. However, the recommendation remains still valid: older adults should meet the micronutrient requirements determined for adults by consuming a balanced diet and micronutrient supplements should only be used when food intake is too low. Neither the European Commission nor the American Academy of Nutrition and Dietetics has suggested a beneficial effect of higher micronutrient reference values for older adults. Furthermore, both have stressed the importance of following the Mediterranean Diet as a whole diet approach to promote healthy aging [313, 328].

Strategies with oral nutritional support are important to deal with established malnutrition or with individuals at risk of malnutrition. In this context, the first step should be dietary modifications to increase the intake of protein and energy from food, while preserving the enjoyment of eating. Oral nutrition supplements (ONS) may be used as adjuncts to the nutritional management. The main measures to increase protein and energy intake include [314, 329, 330]: 1) increasing eating frequency with at least three meals a day and snacks between meals, 2) increasing nutrient density of meals by enriching traditional foods with milk powder, whole milk concentrate, grated cheese, eggs, fresh cream, and nuts, 3) using fortified foods like enriched bread, yogurt, or pasta, 4) drinking nourishing fluids such as milk drinks, smoothies, and juices, 5) prescribing ONS in specific diseases related to malnutrition, eaten as snacks, or added to meals, and 6) developing foods with textures that are adapted to the oral health of elderly consumers.

### Needs for nutrition monitoring

With increasing prevalence of malnutrition among older adults, performing nutritional screening of elderly on a regular basis for the detection of malnutrition risks and early implementation of adequate action plans is essential. These measures will enable older adults to maintain their independence and life in community settings for longer periods and to decrease morbidity and mortality risks associated with malnutrition. Acute diseases are regularly associated with decreased food intake and low physical activity both leading to muscle mass loss, especially in older people. Furthermore, malnourished patients tend to have longer hospitalization times and are more susceptible to infections [331] (see also Part 3). Assessing the risk of malnutrition and implementing a nutritional support during hospital stays is thus essential, along with an adequate rehabilitation program, in order to prevent loss in lean body mass [332]. Finally, nutritional interventions should be continued after discharge from the hospital to maintain independence [333].

The screening of malnutrition must be carried out by primary care doctors at least once a year in general practice and on admission to care homes and hospital [314]. Clinical decision making and implementation of nutritional support relies on objective classification of a patient state, *i.e*. at risk of malnutrition, malnourished or severely malnourished. Simple, validated, and reliable screening tools should be used, as already described in Section 1.3.2. However, as different screening tools may lead to different diagnosis, the assessment instrument must be standardized and used in all screening situations. With appropriate adjustment of the cut-off point for different populations, the MNA and MNA-SF have been extensively used and validated worldwide [334]. The MNA also appears to be the most appropriate tool for nutritional assessment of the elderly living in their homes [335]. If possible, a self-MNA may be used, being even more sensitive and specific in identification of malnutrition when completed by the elderly instead of the caregivers or a health care professionals [336].

Treatment of malnutrition should involve a multidisciplinary team including geriatricians, dietitians, caregivers, and other health professionals when necessary. It should be based on treatment of the underlying cause of malnutrition and on improvement of the nutritional status, the nutritional intervention lasting at least three months [333]. Training, communication, and coordination of nutritional intervention and monitoring between health care professionals in hospital, the community, and caregivers are also essential. Routine monitoring of weight changes, re-evaluation of malnutrition risks, and estimation of food intake are key aspects to assess effects of intervention [314, 333, 334]. Food diaries kept by independent elderly at risk of malnutrition may also be useful to assess nutrient intake and to follow up the implemented dietary plans [337]. Nutritional management may be complemented by devices developed specifically for autonomous elderly. In that regard, pilot studies with telemedical systems [338] and ambient intelligent systems [339] have shown that these devices may promote a more effective nutritional management. Although the elderly users defined these systems as easy to use and appreciated the offering of menus, 50% of them stated that they do not want to be controlled and surrounded by technology, pointing to limitations in the implementation of these devices [339].

### Impact of major food products on malnutrition

The food products building a balanced and correct human diet already contain the nutrients necessary to satisfy the nutritional needs of healthy adults and could significantly counteract the negative effect of malnutrition in elderly. Section 4.4 describes the evidence available for the effectiveness of five major food groups, *i.e*. dairy products, meat products, fish, cereal-based foods, and fruits and vegetables. Nevertheless, in addition to differences in the nutritional needs, the functionality of the GIT and in the food choices should be carefully considered in the formulation and manufacturing of products specifically devoted to elderly. Such a strategy could represent an important step ahead in the counteraction of malnutrition in aged people.

#### Dairy products: proteins, calcium, vitamins, and bacteria

The impact of the ingestion of a range of dairy products on an equally broad range of health-related parameters in elderly subjects has been investigated quite intensively in observational and interventional studies. Regarding the overall nutritional status, an increased consumption of dairy products in elderly correlated with increased intake of energy, protein, vitamins (A, B2, B5, B9, B12), minerals (calcium, magnesium, zinc, phosphorus), and cholesterol [340–346]. Further, some important nutrients, in particular vitamin B9 [345] and vitamin D [346], can be delivered to consumers by fortifying milk.

The ability of dairy products to positively influence the immune system is the health benefit mostly reported for these products in elderly. Most of the evidence was obtained with dairy products containing bacteria, in particular milk fermented to yogurt in the presence of probiotic strains and/or lactic acid bacteria. A positive effect has been shown in a range of clinical indications, such as common cold [347], infectious diseases of the airways including upper respiratory tract infections and rhinopharyngitis [348, 349], common infections in hospitalized patients [340], and infections with *Clostridium difficile* [350]. In addition, an anti-inflammatory activity mediated by products of milk fermentation was identified after consumption of a probiotic yogurt [351], accompanied by a decrease in mutagenicity in the intestinal tract [352]. Dairy products were also reported to strengthen vaccination protocols as shown for influenza vaccination [353, 354]. Another study provided evidence for an immunological contribution of non-bacterial components of dairy products as whey proteins enhanced the serum response of elderly to pneumococcal vaccine [355]. Taken together, dairy products can be considered as strategic food vectors to deliver immunomodulatory components to elderly helping them to strengthen their immune system. Clearly, these findings must be discussed in the context of the important role of the human gut microbiota on the immune system and of the role of nutrition in modulating these interactions [356].

Milk is a key food to sustain bone health, a property that is primarily mediated by calcium, inorganic phosphate, vitamin D, and proteins [357]. A significant positive correlation was observed between dairy nutrient consumption and bone mineral density at the total hip and femoral neck in elderly men, but not in elderly women [358]. Vitamin D-fortified milk also supported the management of hypovitaminosis by increasing 25(OH)D_3_ and calcium levels [346]. Also, vitamin D and calcium-fortified soft plain cheese appeared, at least transiently, to reduce secondary hyperparathyroidism and bone remodeling by decreasing parathyroid hormone and increasing insulin-like growth factor-I (IGF-I) and the bone formation marker P1NP [359]. Another study reported that vitamin D in vitamin D-fortified cheese is bioavailable in both young and older adults although this product was insufficient to increase serum 25(OH)D_3_ during limited sunlight exposure [360]. In women having experienced a recent hip fracture, casein and whey proteins combined with essential amino acids both induced a significant elevation of serum IGF-I [361]. Finally, milk intake positively correlated with serum IGF-I levels in a study involving postmenopausal women enrolled in the Women's Health Initiative [362]. Supplementation of a product composed of essential amino acids and whey proteins with zinc accelerated the serum IGF-I response of elderly to the whey protein while decreasing a biochemical marker of bone resorption [363]. In that context, the European Society for Clinical and Economic Aspects of Osteoporosis and Osteoarthritis (ESCEO) recommended the daily consumption of calcium- and vitamin D-fortified food products (*e.g*. yogurt or milk) by fragile elderly subjects at elevated risk for falls and fracture [364]. Taken together, calcium and proteins in milk, supplemented with vitamins and minerals, in particular vitamin D, mitigate the development of osteoporosis in the elderly.

Dairy products also support muscle health by delivering BCAAs to the aging organism. Milk proteins are rich in the BCAAs leucine, isoleucine, and valine, which, in particular leucine, are required for optimal stimulation of the rate of muscle protein synthesis in the elderly [365], thus preventing age-related sarcopenia. In older men, milk-derived whey proteins stimulated postprandial muscle protein accretion more effectively than caseins. This effect was attributed to the kinetics of digestion and, consequently, intestinal adsorption of whey proteins, which are fastly absorbed compared to the slow caseins. In addition, whey proteins are characterized by a high content of leucine, which further contributes to improving muscle mass [255]. A study evaluating the association of dairy intake with body composition and physical performance in older women revealed significantly greater whole body lean mass and appendicular skeletal muscle mass, greater handgrip strength, and lower odds for a poor Timed Up and Go (TUG) motility test. However, the difference in prevalence of falls was not statistically significant [366]. Although milk protein supplementation combined with low intensity physical exercise in older people suffering from polymyalgia rheumatic tended to prevent accumulation of body fat, it did not result in significant and consistent difference in the changes of muscle mass indices or muscle functions [367]. Finally, milk proteins (casein and whey) have been used as therapeutic modality to conserve muscle mass in chronic wasting diseases such as chronic obstructive pulmonary disease [368, 369].

Other common health problems in elderly may be ameliorated by consumption of dairy products. Yogurt supplemented with dietary fiber relieved constipation [370, 371] and intake of probiotic fermented milk alleviated symptoms of non-viral gastroenteritis [372]. Dental health may be improved by consumption of milk supplemented with fluoride and probiotics [373]. Finally, consumption of yogurt was associated with a lower common carotid artery intima-media thickness in a cohort of Australian elderly subjects [374], suggesting a beneficial effect in CVD risk.

Of note, since the food habits of many elderly people are inadequate, flavor amplification might induce changes in preferences for the consumption of food. Dairy products such as yogurt thus provide an interesting matrix to introduce flavors preferred by elderly in order to more efficiently deliver nutrients tailored to their nutritional needs [375].

#### Meat products: proteins, iron, vitamin B12, and carnosine

Although not strictly necessary in the human diet, meat shows considerable nutritional properties, including a good balance in indispensable amino acids (IAA), and high levels of B vitamins and minerals thus becoming an important component of a balanced diet. In moderate amounts, and combined with other foods being part of a healthy diet (vegetables, starches, fruits, dairy products), meat is useful to prevent possible deficiencies in vitamin B12, iron, zinc, and selenium [376].

In contrast to the general belief of elderly, their protein requirement is not decreased, but rather increased, despite a general decrease in their physical activity [377]. Although meat could efficiently cover their protein requirement, elderly tend to significantly decrease its consumption for multiple reasons (loss of sense of smell and taste, chewing difficulties…). In light of the ability of meat to efficiently deliver IAA, efforts should be made to develop meat products with adapted texture, taste, and digestibility to make them more attractive for the elderly.

Mainly in the form of heme iron, meat iron has a greater bioavailability than non-heme iron of plants or dairy products [378]. Also, heme iron absorption, in contrast to non-heme iron, is largely uninfluenced by other dietary constituents. In addition, meat enhances non-heme iron absorption [379]. Thereby, vegetarian populations in comparison to omnivore populations are generally characterized by a lower plasmatic concentration of ferritin, which is indicative for reduced iron stores [380]. Furthermore, ruminant meat is an important source of vitamin B12. In ruminants, this vitamin, which is strictly of microbial origin, is synthesized in the rumen before being absorbed and stored in the liver and in muscle. For this reason, the vitamin B12 level in ruminant meat is higher than that measured in meat from pork and chicken. Based on average beef consumption, ruminant meat is considered to cover approximately two thirds of the daily intake of vitamin B12 in humans. In addition, the chemical forms of vitamin B12 present in meat are the biologically active forms. This is, for example, not the case for sea foods, which contain high levels of biologically inactive forms of vitamin B12 [381]. An insufficient meat consumption may thus increase the risk of iron and vitamin B12 (sub-) deficiency in humans leading to anemia. It should finally be noted that both heme-iron and vitamin B12 concentrations in meat, can be significantly affected by the technological processes involved in meat preparation [382, 383].

A major feature of aging is the progressive decline in muscle mass, leading to sarcopenia. At an advanced stage, aging causes frailty and a progressive loss of autonomy of the individual. Associated with maintaining regular physical activity, protein nutrition can slow down the development of aging. The mechanisms involved in the development of sarcopenia are multiple and multi-factorial, but all of them result in an imbalance between the synthesis and degradation of muscle protein. This imbalance may be partly explained by a decrease in anabolic response related to food intake. Indeed, in the elderly, the installation of an ‘anabolic resistance’ to the effects of nutrients and hormones translates into a higher threshold of stimulation of the muscle protein anabolism [384]. Noteworthy, it is still possible to trigger protein anabolism in elderly, but with a higher intake of stimuli. Thus, in order to recover, or at least improve, postprandial muscle anabolism in the elderly, it is necessary either to restore the protein metabolism sensitivity to postprandial signals, and therefore to lower the stimulation threshold, or to optimize the postprandial kinetics of amino acids absorption, so as to exceed the stimulation threshold. For the latter option, two nutritional strategies are possible without increasing the overall protein intake, namely concentrating daily protein intake in one meal [325, 385] or using rapidly digested proteins [386]. Because of its high density in protein, rich in IAA, and because it is rapidly digested in healthy elderly [95], meat is a good candidate for the implementation of both strategies. Recent work highlights the importance of meat in the diet of older people to boost muscle protein synthesis [387]. At equivalent amounts of protein, meat was more effective than a source of vegetable protein, such as soybeans, which is rather well balanced in IAA [388]. However, a significant reduction in masticatory efficiency, accompanied by a swallowing of less disintegrated pieces of meat, as frequently observed in advanced age, could compromise its strengths in elderly nutrition. Indeed, edentulous subjects wearing complete dentures had a significantly reduced protein digestion rate and, possibly, digestibility in the small intestine. This has harmful consequences for the elderly as this phenomenon is accompanied by a lower utilization of meat-derived amino acids for the synthesis of body proteins [95]. However, the technological processing of meat (mincing, cooking conditions) could significantly affect meat protein digestion rate, and protein assimilation [389, 390], and thus offer solutions to counteract the chewing inefficiency problems.

Carnosine (β-alanine-L-histidine) and anserine (β-alanine-L-1-methyl-histidine) are histidine-containing dipeptides (HDP) present exclusively in animal tissues. They are particularly abundant in the skeletal muscle of mammals, and thus in meat. Their total amount and proportion may vary from one species to another as well as between muscles. Their concentration in meat is generally only moderately affected by the technological processes [391]. Besides their buffering capacity, HDP also have antioxidant properties towards proteins and nucleic acids, in particular because of their capacity to bind divalent metal ions and trap free radicals. Moreover, HDP appear to reduce aldehydes formed from unsaturated fatty acids during oxidative stress [392]. They may also have a major role in protection against protein glycation and crosslinking [393]. The crosslink of proteins interferes with their tissue function and may lead to aggregation of cell material in the form of plates. Thus HDP could play an important role in the prevention of secondary diabetic complications [394] and in the protection against neurodegenerative disorders such as Alzheimer's disease [395]. Because of these antioxidant properties, diets rich in HDP, in particular meat products, may be beneficial for the elderly [396]. In that regard, a recent study with HDP supplementation in elderly showed promising effect on cognitive functioning [397], with a dose of HDP corresponding to about two meat portions of 120 g/d.

#### Fish: polyunsaturated fatty acids and selenium

Although fish consumption has not been directly used for treatment or prevention of malnutrition, it has been associated with protection of several diseases in elderly, which may either be risk factors or consequences of malnutrition. Health effects of fish consumption have been attributed to the anti-inflammatory properties of n-3 LC-PUFAs, in particular EPA and DHA, which are present in high amounts in oily fish [398]. In the majority of nutritional cohorts investigating the relationship between fish consumption and cognitive function, consumption of one portion of fish per week was associated with a reduced risk for cognitive deterioration, either through association with a healthy eating pattern or through a direct impact of fish consumption [399, 400]. However, elderly consume insufficient amounts of fish and malnourished elderly tend to consume fatty fishes of poor nutritional quality. Nevertheless, the health effects of fish might involve a synergistic effect between selenium and n-3 LC-PUFAs [401]. For over a decade, studies have shown an inverse association of fish consumption with cognitive decline and the development of neurodegenerative diseases in older adults [402–405]. Baierle and collaborators reported that higher plasma levels of total long chain n-3 fatty acids and DHA, and lower levels of saturated fatty acids, were related to a better cognitive performance in older adults [405]. Studies with fish oil or EPA and DHA supplementation have shown memory improvement in elderly with mild cognitive impairment [406, 407]. Other studies with elderly reported that fish consumption reduces CVD markers [405, 408], lowers risk of hip fractures [409], improves kidney function [410], and alleviates depression symptoms [411]. Regarding GIT dysfunction in aging, an inverse relation of periodontal disease progression with dietary DHA intake has been reported in Niigata citizens [412].

Plasma levels of EPA and DHA are positively associated with fish frequency consumption in older people [413]. However, the authors observed that the increase in DHA levels with fish consumption was lower in elderly with APOE4 genotype (main genetic risk factor for Alzheimer's disease characterized by a reduced activity of lipoprotein lipase and increased oxidative stress). These results suggest that bioavailability of n-3 LC-PUFAs may be affected by genetic factors and that this issue must be addressed when designing foods enriched in these fatty acids.

#### Cereal-based foods: carbohydrates and dietary fiber

Carbohydrates have special significance in cereals, which usually comprise about 50–80% carbohydrate on a dry weight basis [414]. Starch is the most abundant cereal polysaccharide being an important energy source in human diets. There are two types of carbohydrates: available carbohydrates, such as starch and soluble sugars, are digested and absorbed by humans, whereas unavailable carbohydrates (dietary fiber), such as resistant starch, cellulose, and other complex polysaccharides (β-glucan, pectins, arabinoxylans…), are not digested by the human GIT.

In 2010, the EFSA established an adequate intake of dietary fiber of 25 g per day for normal laxation in adults based on the role of dietary fiber in bowel function [317]. Moreover, the EFSA approved several health claims related to the consumption of dietary fiber: 1) arabinoxylan contributes to a reduction in postprandial blood glucose, 2) barley grain fiber, oat grain fiber, and sugar beet fiber contribute to an increase in faecal bulk, 3) wheat bran fiber contributes to an acceleration of intestinal transit and increases faecal bulk [415, 416]. Of note, these claims can only be used for foods that are enriched in the particular dietary fiber referred to in the claims. Also, “high fiber”, as listed in the Annex to Regulation (EC) No 1924/2006, indicates that the product contains at least 6 g of fiber per 100 g or at least 3 g of fiber per 100 kcal. In the USA, the Food and Drug Administration (FDA) has approved two health claims for dietary fiber. The first claim states that, along with a decreased consumption of fats (< 30% of calories), an increased consumption of dietary fiber from fruits, vegetables, and whole grains may reduce some types of cancer [417]. “Increased consumption” is defined as ≥ 1 “ounce equivalent” (1 ounce = 28.3 g), with three ounces derived from whole grains. A one ounce equivalent would be consistent with one slice of bread, ½ cup oatmeal or rice (1 cup = 237 ml), or five to seven crackers. The second FDA claim supporting health benefits of dietary fiber states that diets low in saturated fat (< 10% of calories) and cholesterol as well as high in fruits, vegetables, and whole grain, decrease the risk of coronary heart disease [418]. In summary, an healthy diet should be composed of 25–35 g dietary fiber per day, of which 6 g should be soluble fiber [419]

During the last decades, epidemiological and clinical studies demonstrated that consumption of dietary fiber and whole grain has a positive impact against obesity [420], type 2 diabetes [421], cancer [422], and CVD [423] in middle aged people. In 535 healthy elderly, aged 60–98 y, diets rich in whole-grain foods appeared to delay the onset of the metabolic syndrome [424]. In a recent study with mature women (50–72 y) the intake of a diet rich in fiber (43–47 g/d) during 4 weeks decreased serum total cholesterol, LDL-cholesterol, and to a lower extent, HDL-cholesterol [425]. The mechanism of action of the beneficial effects of dietary fiber and the bioactive components mediating these effects are still unclear. Among these, the protective effects of short-chain fatty acids, such as propionate, obtained by the fermentation of dietary fiber in the gut [426], an increased rate of bile excretion reducing serum cholesterol [427], and a decrease in pro-inflammatory mediators, such as interleukin-18 (IL-18) and C-reactive protein (CRP) [428, 429], should be highlighted. In summary, data on the impact of dietary fiber on the health of elderly are scarce or, even, non-existent regarding the specific needs of malnourished elderly.

Within cereal products, bread is one of the most popular food products. It is part of a majority of diets consumed around the world although bread products and their preparation techniques vary widely [430]. The basic ingredients are cereal flour, water, yeast or other fermentative agents, and salt [431]. However, optional ingredients can be added to improve processing or to produce speciality and novelty breads, which often have an increased nutritional value. Bread provides essential nutrients such as carbohydrates, B vitamins (B1, B2, B3, B6, B9) and minerals (phosphorus, potassium, magnesium). Due to these nutritional properties, nutrition experts define bread as an essential constituent of the food pyramid. In countries where bread is a staple source of carbohydrates, it does indeed constitute the base of the diet [432].

#### Fruits and vegetables: phytochemicals

Although increasing evidence on the health effects of phytochemical compounds, particularly polyphenols, has been accumulating for several years, studies with older adults are scarce. Inflammation plays a critical role in the aging process and associated degenerative diseases. Thus, the anti-inflammatory properties of phytochemical compounds are the basis of their beneficial effects on healthy aging [433].

There were few controlled trials studying the impact of consumption of food rich in polyphenols exclusively in the elderly for periods of at least 8 weeks. Most of the studies focused on improvement of cognitive function as a form of prevention or treatment of dementias, which are diseases with increasing prevalence among elderly and one of the risk factors for malnutrition. Several polyphenols or foods enriched in polyphenols have shown improvements in cognition including flavonoid rich cocoa based drink [434], concord grape juice [435], resveratrol [436], and Gingko biloba [437]. Polyphenols have also potential benefits against other diseases associated with malnutrition, such as catechins from green tea extract against sarcopenia [438] and polyphenol-rich chocolate against chronic fatigue syndrome [439]. Red wine (alcohol free) ingestion seemed to facilitate the swallowing reflex in elderly patients with dysphasia, a pathology associated with GIT dysfunction [440].

Some studies with postmenopausal women addressed the effect of isoflavone consumption in the prevention of osteoporosis. It seems that isoflavone supplementation has, on its own, no effect in preventing BMD decline [441]. However, when supplemented with vitamin D, vitamin K1, and PUFAs, isoflavone maintained BMD and increased bone-specific alkaline phosphatase [442]. A recent review has also suggested that flavonoids protect against bone loss by promoting osteoblast function and reducing chronic low-grade inflammation [443].

Assessing their bioaccessibility and bioavailability will be a key issue to validate the use of bioactive phytochemical compounds to fortify foods for elderly. In particular, a large variability in these parameters may arise from digestion impairment, changes in microbiota composition, and polymorphisms in phase II enzymes [444]. Another important issue is the impact of food processing on the bioaccessibility and bioavailability of polyphenols. For example, quercetin in cereal bars seems to be more bioavailable than in powder filled capsules [445].

#### Water

Elderly are more vulnerable to water imbalance and many of them do not reach their recommended daily intake of oral fluids [446]. Anorexia and malnutrition tend to worsen fluids intake due to decreased appetite and decline in the motivation to eat or drink. In addition, higher thirst thresholds [447] and adverse effects of drugs, such as diuretics, also contribute to dehydration [448].

In the absence of fluid intake death occurs in a few days to a week, thus far more quickly than in the absence of any other nutrient [449]. Suboptimal hydration in elderly is associated with increased susceptibility to urinary tract infections, pneumonia, pressure ulcers, confusion and disorientation, whereas adequate hydration is associated with fewer falls, lower rates of constipation, better rehabilitation outcomes in orthopedic patients, and reduced risk of bladder cancer in men [450].

The best preventive measure to reduce dehydration risk in elderly is to ensure that the elderly person has a fluid intake of at least 1.7 L in a period of 24 h, with an additional intake of 500 mL per degree above 38°C when fever is present [451]. Several strategies to increase fluid intake in elderly have been recently reviewed, however, most failed to be conclusive [9]. When hospitalized or residing in care homes, the most efficacious measures seem to be encouragement and assistance from staff to drink more liquids during meals, between meals, and when taking medication. Elderly with dysphasia problems should be given thickened drinks. Availability of beverages and greater choice is also an important factor to increase fluid intake in elderly [9]. Adequate choices include mineral drinks, fruit and vegetable juices, milk, and sport drinks [451]. Independent elderly living at home should be provided with counseling on the amount of fluid intake and the best choices of drinks.

## CONCLUSIONS

A wealth of information is available on the aging process and on the consequences of this process on the functionality of the organ systems in humans. Interestingly, although malnutrition is recognized as an important morbidity factor in the elderly, this phenomenon has not been systematically analyzed in the context of the aging GIT. The major deficiencies in nutrients that are associated with malnutrition have been already identified (energy, amino acids, vitamins, minerals…) and dietary recommendations, including recommendations for the consumption of a range of major food groups covering most of the macronutrients and micronutrients needed by elderly populations, are already available from institutional bodies, thus contributing to limiting the impact of malnutrition on the health status of elderly. These efforts are, however, far from having reached their promises, not the less because the knowledge covering the efficiency of the delivery, by the aging GIT, of nutrients to the organism has not be integrated neither into the health status of the elderly nor into the formulation of new foods tailored on the elderly. Consequently, guidelines proposing optimal diets for elderly populations are as good as the heterogeneity of the malnutrition status of the populations and individuals that they target.

A new paradigm must therefore be developed in order to more efficiently prevent and treat malnutrition in the elderly. This review shows that most of the relevant mechanistic and physiological information is already available to move in that direction. However, each of these elements needs to be integrated into a global concept that encompasses the molecular composition of foods, the processing of the nutrients by the aging GIT, and the impact of the bioavailable nutrients on the health status, in particular the nutritional status of elderly. Recent breakthroughs in molecular analytical sciences now allow for a more efficient search for specific biomarkers in foods (foodomics) and humans (nutrigenomics). These technologies should be used to deliver, not only biomarkers of food intake [452], but, more specifically, biomarkers of food bioavailability reflecting the specific functional changes taking place in the aging GIT. In addition, the same analytical strategy should be used to identify biomarkers of malnutrition [453] in the elderly. An integrated and correlative analysis of both sets of biomarkers (*i.e*. bioavailability and malnutrition) should be conducted at the level of individual elderly subjects to allow for an early identification of decreased functionalities in their GIT and to more precisely identify dietary solutions able to restore the deficient metabolic profile associated with their particular nutritional status. Finally, the ability of specific foods to efficiently deliver nutrients to the organism should be screened *in vitro* in models of food digestion [454] that should be further validated to model the aging GIT. Although these goals are ambitious, the social and economic burden of malnutrition in the elderly provides sufficient motivation to use the information summarized in this review to start paving the road in that direction.

## Abbreviations

ALA: α-linolenic acid
AMD: age-related macular degeneration
ARA: arachidonic acid
BCAA: branched-chain amino acid
BMD: bone mineral density
CCK: cholecystokinin
CVD: cardiovascular disease
DHA: docosahexaenoic acid
EFA: essential fatty acid
EFSA: European Food Safety Authority
EGFR: epithelial growth factor receptor
EPA: eicosapentaenoic acid
GIT: gastrointestinal tract
GIP: glucose-dependent insulinotropic polypeptide
GLP1: glucagon-like peptide 1
GLP2: glucagon-like peptide 2
HDP: histidine-containing dipeptide
IAA: indispensable amino acid
LA: linoleic acid
LC-PUFA: long-chain PUFA
IGF-I: insulin-like growth factor-I
MNA: mini nutritional assessment
MNA-SF: MNA short form
PYY: peptide YY
PUFA: polyunsaturated fatty acid
RDA: recommended dietary allowance
ROS: reactive oxygen species
VDR: vitamin D receptor
1,25(OH)2D_3_: 1α,25-dihydroxyvitamin D_3_
25(OH)D_3_: 25-hydroxyvitamin D_3_
